# Policy, practice, and prediction: model-based approaches to evaluating N. *gonorrhoeae* antibiotic susceptibility test uptake in Australia

**DOI:** 10.1186/s12879-024-09393-y

**Published:** 2024-05-17

**Authors:** Phu Cong Do, Yibeltal Alemu Assefa, Suliasi Mekerusa Batikawai, Megbaru Alemu Abate, Simon Andrew Reid

**Affiliations:** 1 School of Public Health, The University of Queensland, Herston, QLD Australia; 2 Department of Medical Laboratory Science, Bahir Dar University, Bahir Dar, Ethiopia

## Abstract

**Background:**

Antimicrobial resistance (AMR) represents a significant threat to global health with Neisseria *gonorrhoea* emerging as a key pathogen of concern. In Australia, the Australian Gonococcal Surveillance Program (AGSP) plays a critical role in monitoring resistance patterns. However, antibiotic susceptibility test (AST) uptake – a crucial component for effective resistance surveillance – remains to be a limiting factor. The study aims to model the processes involved in generating AST tests for N. *gonorrhoea* isolates within the Australian healthcare system and assess the potential impact of systematic and policy-level changes.

**Methods:**

Two models were developed. The first model was a mathematical stochastic health systems model (SHSM) and a Bayesian Belief Network (BBN) to simulate the clinician-patient dynamics influencing AST initiation. Key variables were identified through systematic literature review to inform the construction of both models. Scenario analyses were conducted with the modification of model parameters.

**Results:**

The SHSM and BBN highlighted clinician education and the use of clinical support tools as effective strategies to improve AST. Scenario analysis further identified adherence to guidelines and changes in patient-level factors, such as persistence of symptoms and high-risk behaviours, as significant determinants. Both models supported the notion of mandated testing to achieve higher AST initiation rates but with considerations necessary regarding practicality, laboratory constraints, and culture failure rate.

**Conclusion:**

The study fundamentally demonstrates a novel approach to conceptualising the patient-clinician dynamic within AMR testing utilising a model-based approach. It suggests targeted interventions to educational, support tools, and legislative framework as feasible strategies to improve AST initiation rates. However, the research fundamentally highlights substantial research gaps in the underlying understanding of AMR.

**Supplementary Information:**

The online version contains supplementary material available at 10.1186/s12879-024-09393-y.

## Introduction

Antimicrobial resistance (AMR) is an emerging global health crisis which poses significant threat to public health systems [1]. Of particular interest is the sexually transmitted infection (STI) caused by Neisseria *gonorrhoeae* [2]. N. *gonorrhoeae* is a STI which has emerged as a bacterium of particular interest with the rapid emergence of multi-drug resistant species [3, 4]. As an implication for public health systems, a thorough understanding of N. *gonorrhoea* epidemiology is imperative for the evaluative processes involved in refining stewardship efforts [2, 5, 6].

In the context of Australia, the development and implementation of the Australian Gonococcal Surveillance program (AGSP) signalled the significance of understanding N. *gonorrhoea* epidemiology and shifts in resistance patterns [7]. In synergy with legislation mandating the notification of N. *gonorrhoeae* infection, the AGSP operates by collating data from Australian state and territory reference laboratories [7]. For resistance data to be collected along with the notification, the isolate must have an antibiotic susceptibility test (AST) performed [7]. The processes involved in the system ultimately aim to inform clinical management and public health policies. However, there are significant challenges in understanding resistance patterns with the increasing use of molecular-based diagnostic testing methods like that of nucleic acid amplification tests (NAAT) limiting the availability of isolate resistance data [8].

Australia’s AMR strategy is currently rudimentary and without strategic objectives [9–11]. Previous efforts to evaluate the AMR surveillance system has found clinician initiation of ASTs as the most influential factor to delineation of strategic objectives [12]. Fundamentally, the AGSP requires associated ASTs to be present with from N. *gonorrhoeae* isolates to enumerate data [7]. In endeavour to delineate strategic objectives, the absence of isolates limits the capability for robust epidemiological estimates of N. *gonorrhoeae* prevalence and resistance patterns to be enumerated. This absence of empirical data to guide stewardship limits the generation of effective stewardship efforts.

Model-based approaches offer a structured paradigm to understanding the intricacies of a healthcare system and its outcome within a given context. The employment of modelling presents the inherent benefits of identifying the underlying factors and quantifying their relationships to facilitate a greater comprehension of the system’s dynamics. Within wider AMR literature, models have been developed to evaluate vaccination programs, identify economic opportunities, and better understand transmission dynamics [13]. A similar approach can be taken to understand the determinants of AST initiation and identify potential scenarios for which improvements can be made.

### Study aims and rationale

The following paper aims to provide a foundation for future stewardship efforts by quantitatively modelling the processes involved within AST generation and the impact of systematic changes to the proportion of ASTs initiated. The rationale for understanding the determinants of AST initiation is pivotal for improving objectives set out by the AGSP. Identifying and modelling the interactions between factors allows for a greater comprehension of the current system. The study is informed by 2 key objectives:

1. The identification and modelling of key factors implicated in the AST generation process.
2. The assessment of potential impact of various systematic and policy-level interventions on the rates of AST initiation.

## Methods and methodology

### Modelling methodology

The study aims to model the clinical and diagnostic processes involved in the initiation of AST for N. *gonorrhoeae* isolates. The complexity of the clinician-patient interaction with variability in diagnostic and clinical practices substantiate difficulty in a definitive representation. For the generation of a data point in the AGSP regarding resistance, an isolate must receive have an AST initiated to determine resistance status. Despite the given complexities, the following models aim to simulate the processes to better improve the data generation processes of the AGSP.

Two models have been developed to represent the processes involved in the generation of AST for N. *gonorrhoea* isolates. Two paradigms have been presented in the absence of detailed patient-clinician dynamics The models serve to explore different hypotheses concerning the structure. The first model is a mathematical stochastic health systems (SHSM) model. The second model selected is a Bayesian Belief Network (BBN).

#### Identification and selection of variables

In the process of constructing the models, variables for each model had to be identified. A systematic search of academic literature was conducted. The purpose of the search was to identify the main determinants that are implicated within the clinician-patient dynamic that was to be modelled. In acknowledging the absence of literature directly regarding N. *gonorrhoea* AST tests and its determinants, the search was widened to general determinants in sexually transmitted infection (STI) diagnoses. Following the search, two reviewers PD an SB independently read through the search results and identified key barriers and facilitators to initiation of diagnostic testing. Following the extraction of literature, an influence matrix was created to represent the causal relationships between the identified variables. All details of the systematic search, identification of variables, and influence matrix are included in supplementary file 1.

#### Stochastic health systems model and outputs

**Fig. 1:**
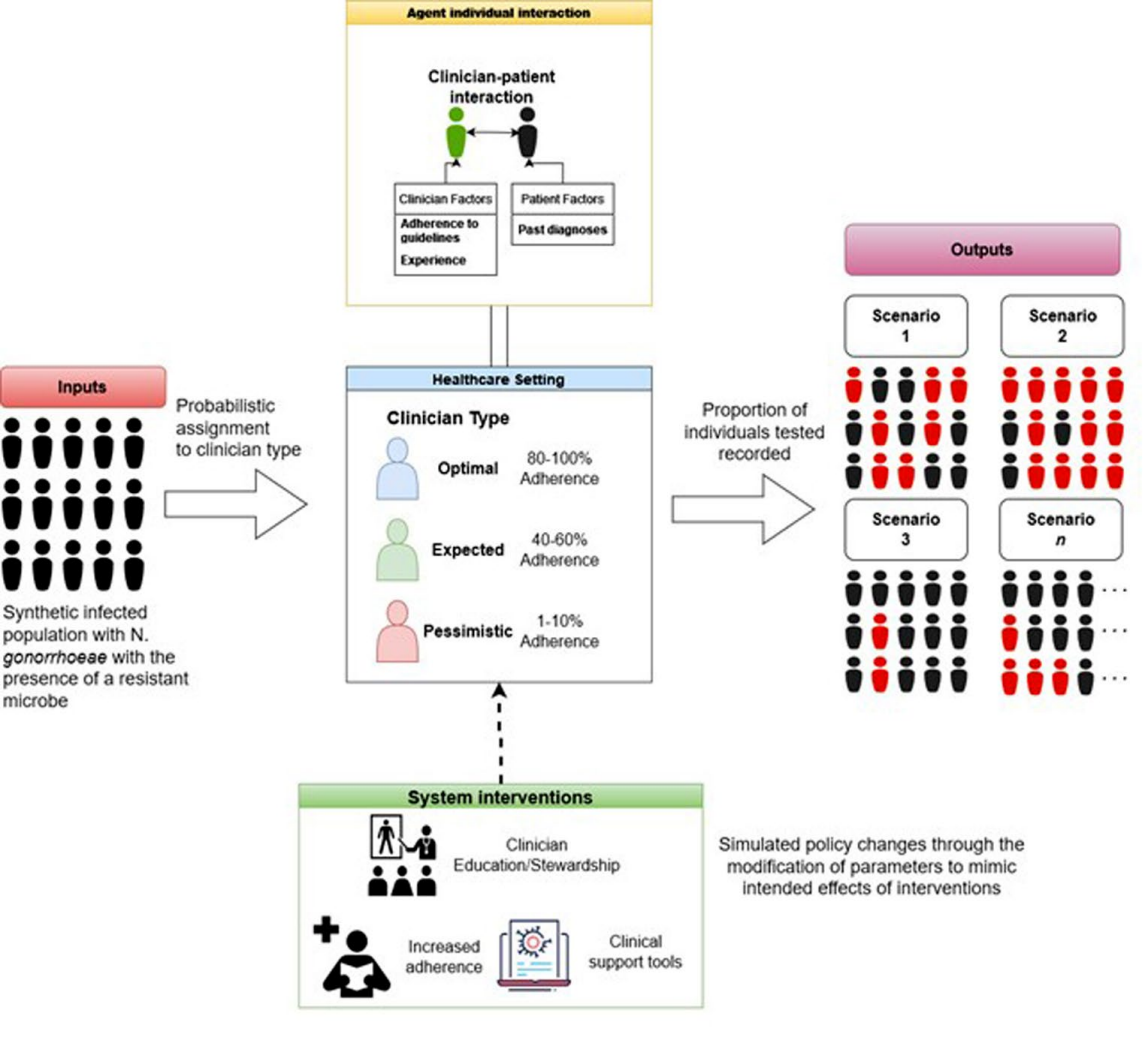
Schematic representation of the stochastic health systems model for N. *gonorrhoea* antibiotic susceptibility testing with proposed interventions and outputs

The stochastic health system model (SHSM) is a mathematical model which represents a paradigm whereby the AST initiation appear random at a systems level. Broadly defining the paradigm, the health systems level refers to the overarching structures and processes that govern the health service at a population level. While random, the model outputs are influenced by a multitude of deterministic factors within the patient clinician dynamic that collectively drive the probability of AST initiation. The structure of the model allows for the exploration of system-level modification of factors which may shape the probability of testing. The schematics of the stochastic health systems model and the proposed outputs and interventions are depicted in Fig. 1.

The model operationalises the process of N. *gonorrhoea* AST testing by generating a synthetic population with confirmed cases of N. *gonorrhoea* that exhibit resistance. All parameters for the modelling process are available in supplementary material 2. The stochastic health system’s model was created and implemented within R (Version 4.2.3). At baseline, clinician adherence (α) was set to the following proportional split of 70%/25%/5% for moderate, high, and low adherence distributions. All population parameters and inputs are available in supplementary file 2 (Table S1–S4).

The model works by procedurally generating a synthetic population $\left(n=\text{10,000}\right)$, modelled to population parameters, which then interacts with the clinicians to simulate entering the health system. The model is run 10,000 times and produces a mean value with 95% confidence intervals. The patient-clinician interaction is modelled as the product of clinician knowledge and patient risk which would result in the initiation of an AST. Following the generation of the synthetic population based on the specified parameters, a probabilistic assignment to clinician type is completed. The probability for assignment denotes the proportion of agents available which is defined as $\text{Pr}\left({a}_{i}\right)$ where ${A}_{i}$ would be the population assigned to agent ${a}_{i}$.

$${A}_{i}=n\cdot \text{Pr}\left({\text{a}}_{\text{i}}\right)$$

The clinician patient interaction models clinician and patient factors through a weighted summation of risk. Program evaluation and review technique (PERT) distributions have been used to parameterise odds ratios derived from literature and implement stochasticity. Supplementary file 2 (Table S2–S4) contains all the parameter values. Patient risk θ_*i*_ and clinician experience τ_*i*_ are quantified by aggregating binary variables ${x}_{i}$ for patients and ${y}_{i}$ for clinicians multiplied by their respective weights ${w}_{i}$ and ${z}_{i}$.

$${{\theta }}_{i}\left({x}_{i},{w}_{i}\right)={\sum }_{i=1}^{n}{x}_{i}\cdot {w}_{i} \hspace{1em}{\tau }\left({y}_{i},{z}_{i}\right)={\sum }_{i=1}^{n}{y}_{i}\cdot {z}_{i}$$

In our model, we normalise both patient risk and clinician experience scores to a $\left[\text{0,1}\right]$ interval using sigmoid functions φ_*a*_ for patients and φ_*b*_ for clinicians. These sigmoid functions represent the probabilities that influence the initiation of AST. Specifically, φ_*a*_(θ_*i*_) transforms patient score θ_*i*_ into probabilities reflecting likelihood of requiring an AST due to having resistance present. For clinicians φ_*b*_(τ_*i*_) maps clinician test score τ_*i*_ into probabilities reflecting the likelihood of initiating test, modified by adherence parameter α. For example, 70% adherence rate would set α=0.7.

$${{\phi }}_{\text{a}}\left({{\theta }}_{\text{i}}\right)=\frac{1}{1+{\text{e}}^{-{{\theta }}_{\text{i}}}} \hspace{1em}{{\phi }}_{b}\left({{\tau }}_{i}\right)=\frac{1}{1+{e}^{-{\alpha }\cdot {\tau }\left({y}_{i},{z}_{i}\right)}}$$

Finally, to get the probability of an individual is tested such that they are perceived as a risk, λ_*i*_, we model the probability it as two independent events given by the transformed risk probabilities as previously calculated.

$${\lambda }_{i}\text{= Pr}\left(\text{Risk}|\text{Test}\right)={{\phi }}_{a}\cdot {{\phi }}_{b}\hspace{1em}\text{where}\hspace{1em}{{\lambda }}_{\text{i}}\in \left[\text{0,1}\right]$$

Then test status is then assigned using the binomial function ${{\phi }}_{i}$ with the probability of success being ${{\lambda }}_{i}$.

$${{\phi }}_{i}\sim \text{Binomial}\left(n,{{\lambda }}_{\text{i}}\right)$$

Supplementary file 2 (Fig S9) contains the displays the sensitivity analysis of the binomial function based on probabilities. Intervention scenarios include the improvement of clinician knowledge through stewardship or education, mandates with increased adherence, and the introduction of clinical AMR support tools. Further information regarding the scenario permutations is included in supplementary file 2.

#### Bayesian belief network building

Bayesian Belief Networks (BBNs) are a graphical, probabilistic modelling technique which enables the explicit representation of conditional dependencies between variables and causal pathways, often through a directed acyclic graph (DAG) [14]. BBN comprise of 3 main elements: (1) A set of variables representing a system with a set of states; (2) links between variables to denote causal relationships and influence; (3) Conditional probabilities tables (CPTs) which describe the influence that the variables exert given a denoted relationship [15]. These CPTs facilitate the computation of the joint probability over a set variable using the Bayes’ Theorem to provide a probabilistic output for given a range of states:

$$\text{P}\left({X}_{1},{X}_{2},\dots ,{X}_{n}\right)={\prod }_{i=1}^{n}P\left({X}_{i}|\text{Parents}\left({X}_{i}\right)\right)$$

The BBN has been chosen as it represents a non-random paradigm whereby the initiation of an AST test is seen as the result of conditional interdependencies. Fundamentally, the BBN suggests the probability of AST test initiation by the clinician is solely dependent on the individual’s presentation to the healthcare system. Unlike the stochastic model, the model focuses on individual level determinants rather than viewing it at a systems level.

#### Bayesian belief network parameterisation

The BBN was developed using a two-step approach. The first was a systematic literature search to identify the key structures implicated and processes in initiating an AST. Following the systematic search, a dependency matrix was developed to denote core relationships. The initial model was built within Netica (NorSys) and later implemented in GeNIe (BayesFusion) for validation. Further refinements to the model structure were made using expert elicitation. Due to the absence of empirical data, the conditional probability tables (CPTs) were parameterised using expert opinion. Adapted from Cain [16], for consistent population of the CPTs, the base case scenario and worst case scenario were used as benchmarks for probability allocation.

#### Bayesian belief network evaluation

The model evaluation step focuses on checking the consistency and robustness of the BBN. However, it is to be noted that the metrics used to evaluate predictive capability do not imply a comprehensive causal model. Though, the predictive power is a desirable feature of a causal model. To evaluate the model’s consistency, a sensitivity analysis on key variables to identify the most influential relationships was conducted. Variance of beliefs was used as the main metric to assess the influence of parent nodes on the target node. If needed, adjustments were then made to the model structure to rectify discrepancies in relational weighting of nodes.

To evaluate the robustness of the BBN, the receiver operator curve (ROC) was the main metric used to assess the model’s predictive performance. The model was calibrated using K-fold partitioning $(K=2$) using a generated casefile from GeNIe $(n=\text{10,000})$. The data was partitioned into training and testing datasets using an 80%/20% split respectively. Following the learning of CPTs based on maximisation of expectation using the training data, the initial parameters were then updated by fitting the data file to the final model. The model was then tested on the testing dataset to produce an ROC curve.

#### Core assumptions of both models

The models have underlying assumptions which are critical to the interpretation of their outputs. Primarily, both models presuppose that historical data gathered from the past 15 years (2008–2022) are adequate to represent the continuing trends of proportion of ASTs initiated. Within the stochastic model, this assumption further extends to the randomness at a systems level whereby outcomes are influenced by a nuanced interaction of stochasticity and determinism. Conversely, the BBN is fundamentally predicated on the existence of conditional interdependence of determinants for a test to be initiated.

Additionally, both models implicitly rely on external factors such as resource availability, and policies to be embedded within the model’s parameters without dynamically modelling their temporal impact. Explicit external assumptions within both models assume the following: (1) population homogeneity in the presence in resistance to simplify the patient-client interactions with all resolvable diagnoses; (2) availability of resources with negligible impact due to locality; (3) assumed immediate follow up with AST testing and (4) the mechanism for resistance testing is elective with Nucleic Acid Amplification Testing (NAAT) being the default pathway for N. *gonorrhoea* diagnosis. Further detail regarding the model’s individual parameter assumptions have been listed in supplementary file 2 for the stochastic health systems model and supplementary file 3 for the BBN.

### Parameterisation of historical data

**Fig. 2:**
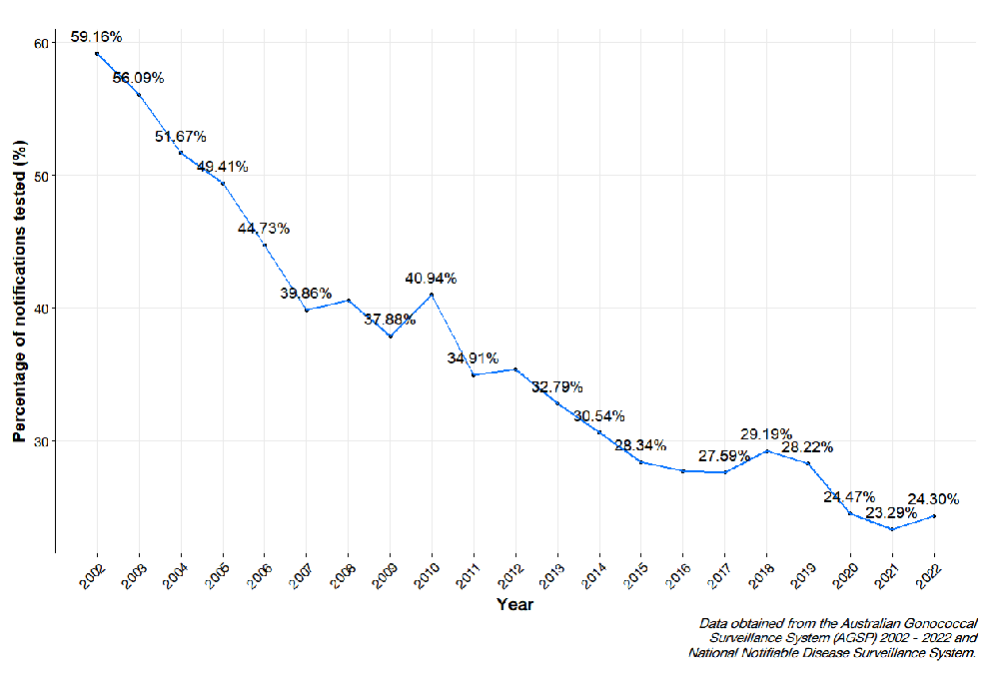
Timeseries depicting the proportion of N. gonorrhoea isolates tested from the Australian Gonococcal Surveillance Program (AGSP) between 2002–2022

The BBN and the stochastic health system model requires the parameterisation of historical data to validate the probabilistic outputs. This approach would allow for a robust basis for comparison between the model outputs and empirical evidence. In parameterisation, historical data was obtained from publicly accessible AGSP reports from 2002 to 2022 to determine the number of isolates tested for the respective year [8, 17–28]. The number of notifications were obtained through extraction of publicly available data from the National Notifiable Diseases Surveillance System (NNDSS) data visualisation tool [29]. The proportion of isolates tested during the period is depicted in Fig. 2.

We adopt a beta distribution to model the variability in proportion of notifications with an isolate. The shape parameters α and β are determined by empirical data, whereby α represents the number of cases with isolates tested. Parameter α is calculated as $k+1$ where $k$ is the number of isolates received during the overall period of 2008–2022. Parameter β is calculated as $n-k+1$ where *n* is the total number of N. *gonorrhoea* notifications within specified period of 2000–2022. Therefore, the proportion of tests initiated, denoted by random variable ${X}_{i}$, can modelled by the following beta distribution:

$${X}_{i}\sim \beta \left(\text{11354,214662}\right)$$

To allow for greater variation in proportions, the parameters α and β were divided by a value of 10. The final distribution used was:

$${X}_{i}\sim \beta \left(\text{1135.4,21466.2}\right)$$

## Results

### Results of the stochastic health system model and the bayesian belief network

**Fig. 3:**
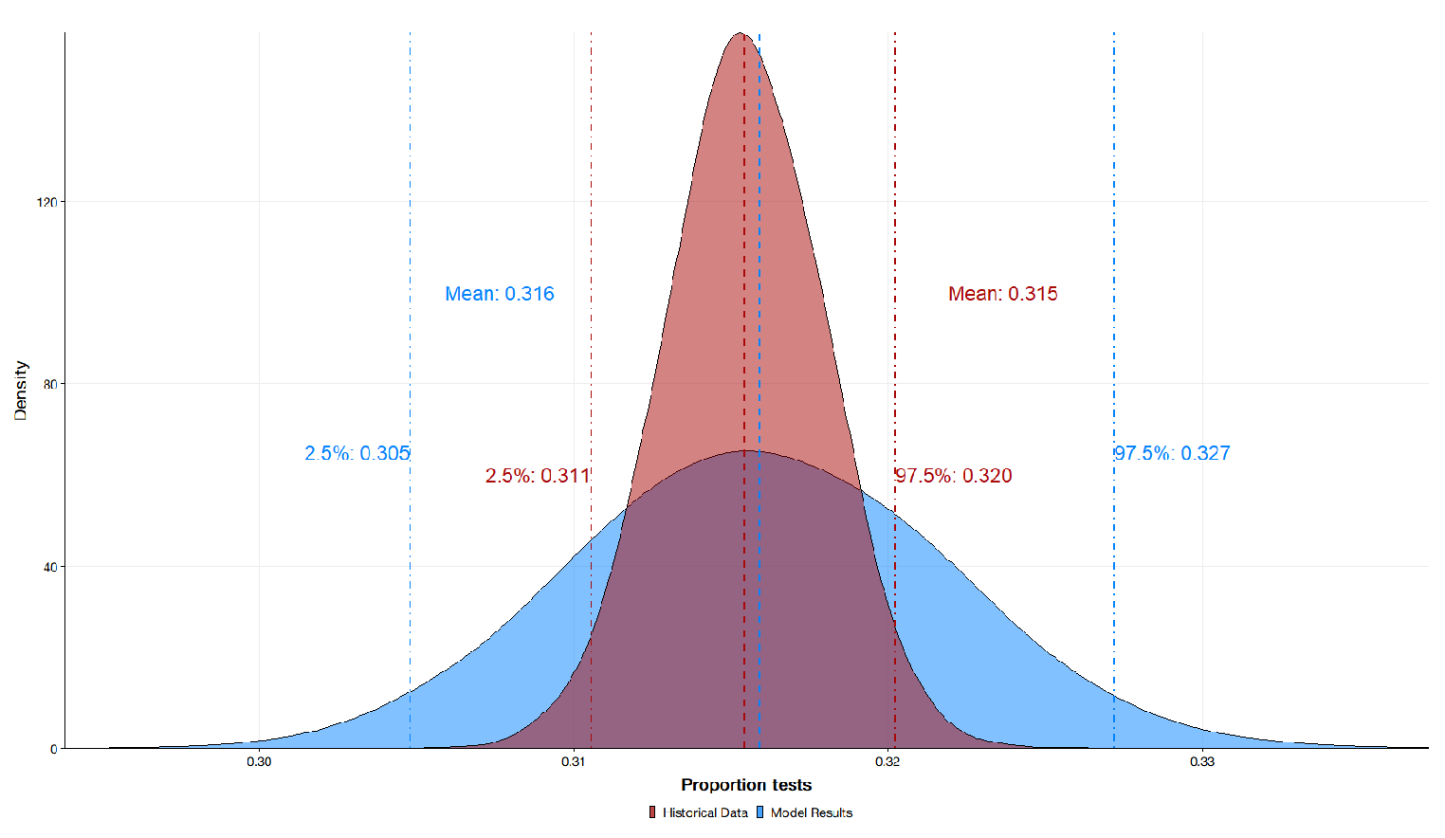
Results of the Stochastic Health Systems Model validated to historical data parameterised by $\beta \left(1135.4, 24166.2\right)$

The stochastic health systems model is presented in Fig. 3. The stochastic health systems model yielded a mean expected test proportion of 0.316 (95% CI; 0.305, 0.327). This test proportion output by the model suggests 31.6% of confirmed N. *gonorrhoeae* cases would have an AST initiated. The outputs of the model are shown to have congruence with the historical data modelled by the beta distribution, $\beta \left(1the.\text{4,21466.2}\right)$ with the expected test proportion of 0.315 (95% CI; 0.311, 0.320). From initial review, the stochastic model demonstrates accuracy in capturing the central tendency of the historical data for the proportion of AST initiated.

**Fig. 4:**
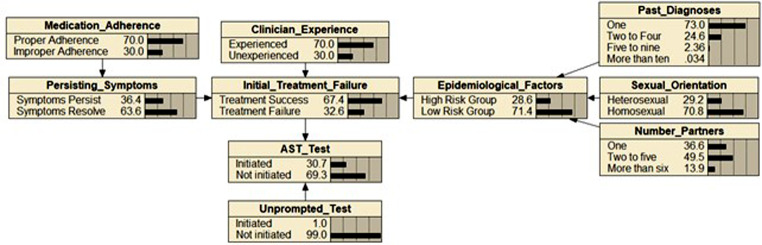
Bayesian Belief Network (BBN) for the initiation of an antibiotic susceptibility test (AST)

The BBN is presented in Fig. 4. The full model with CPTs and the sensitivity analysis is available in supplementary file 2. The BBN developed consists of 10 nodes. These nodes cover the factors of past diagnoses, sexual orientation, number of partners, epidemiological factors, medication adherence, persisting symptoms, clinician experience, initial treatment failure, AST test and unprompted test. Broad categorisations of the model can be made. Epidemiological, factors represent the population level factors inclusive of past diagnoses, sexual orientation, and number of sexual partners an individual presents with. Individual level factors include medication adherence and the persistence of symptoms. Clinical experience of the clinician with sexual health is represented by the clinician experience node. Unprompted tests accounts for the possibility of a test being initiated regardless of the of any factors. The target node of the BBN is AST test. At the initial state, the BBN would indicate most cases of N. *gonorrhoea* would not be tested (69.3%) with the populated epidemiological data and estimated clinician experience.

### Scenario analyses

The results of the scenario analysis for the SHSM and BBN were modelled by the modification of model parameters are presented below. Further details on how the modifications were made can be seen in supplementary file 2 for the SHSM and supplementary file 3 for the BBN.

#### Improvement of clinician knowledge through education or stewardship

**Fig. 5:**
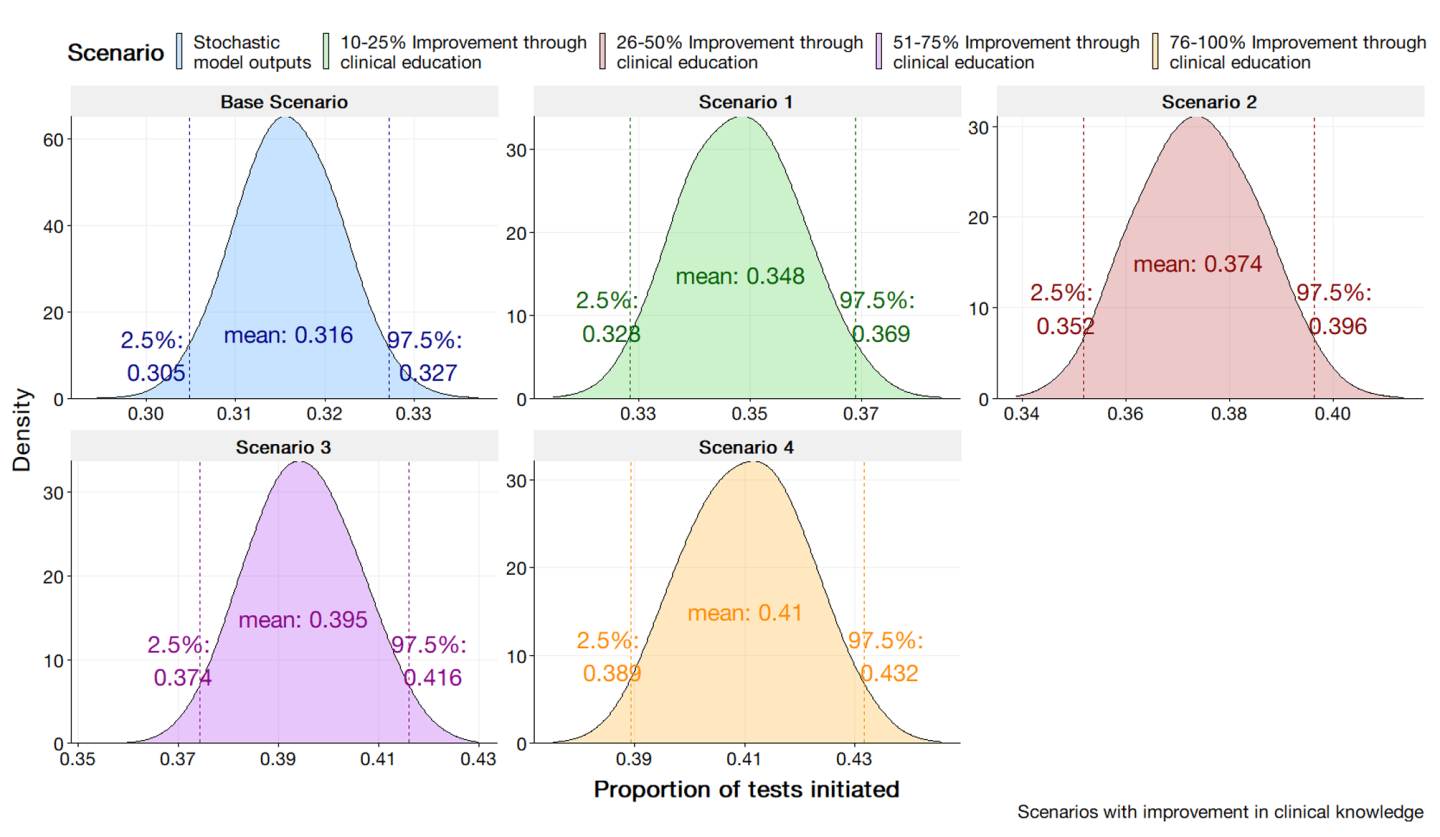
Stochastic health system model outputs in the scenario of improving clinician awareness through education and/or stewardship via the modification of clinician knowledge variable at a split 70%/25%/5% clinician population (average, high, and low adherence)

**Fig. 6:**
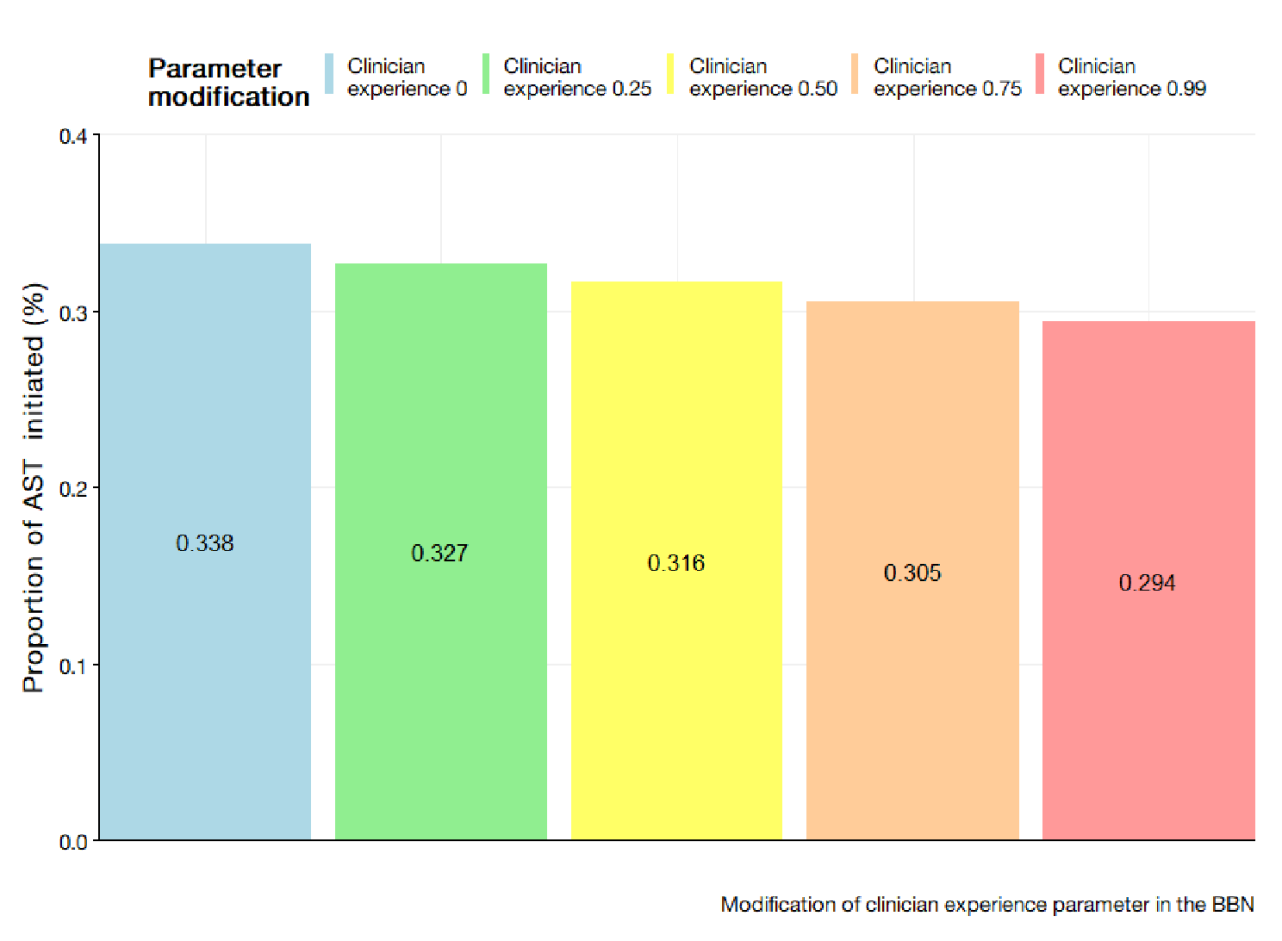
Improvement of clinician experience parameter in the BBN at values of 0, 0.25, 0.50, 0.75, and 1.00

The rationale for this scenario were assumed to have greater awareness regarding resistance of isolates. To mimic an improvement in clinician knowledge in the SHSM, the parameters for clinicians’ experience were incrementally increased by percentages of 10–25%, 26–50%, 51–75%, and 76–100%. For the SHSM, the improvement is assumed to be uniform across the population. The BBN considers the deterministic nature of clinician experience during a consultation. The parameter values for clinician experience were modified at incremental values of 0, 0.25, 0.50, 0.75, and 1.0. The results are displayed in Fig. 5 for the SHSM and Fig. 6 for the BBN.

As a general trend, simulating the improvement of clinician awareness through the modification of the clinician knowledge parameter yielded a linear increase across categories from the base scenario. An incremental increase in clinician knowledge by10-25% yielded an estimate of 0.348 (95%; 0.328, 0.369). A 76-100% increase in clinician knowledge yielded an estimated proportion of tests initiated to be 0.410 (95% CI; 0.389, 0.432).

The modification of clinician experience parameter in the BBN through the increase of values produced lower proportions of AST initiation. The data would indicate a decreasing trend with the modification of the clinician parameter to 0 indicating an increase test proportion at 0.338 when compared to the clinician parameter being set to 1.0 yielding test initiation rates of 0.294.

#### Improvement through the introduction of clinical support tools

**Fig. 7:**
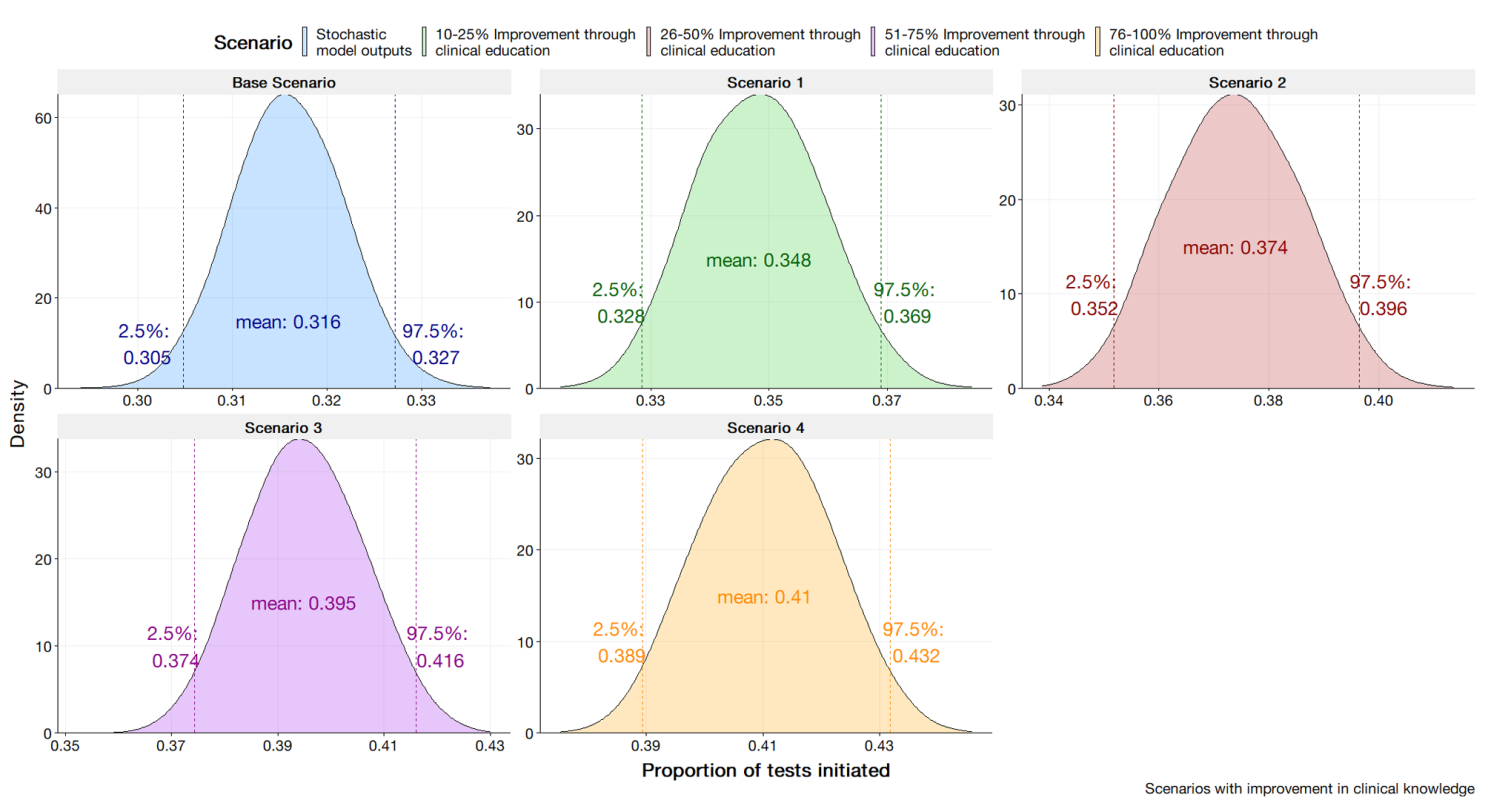
Stochastic health system model outputs in the scenario of introducing clinical support tool to various proportions of clinicians at base estimate of 70/25/5 clinician population (average, high, and low adherence)

**Fig. 8:**
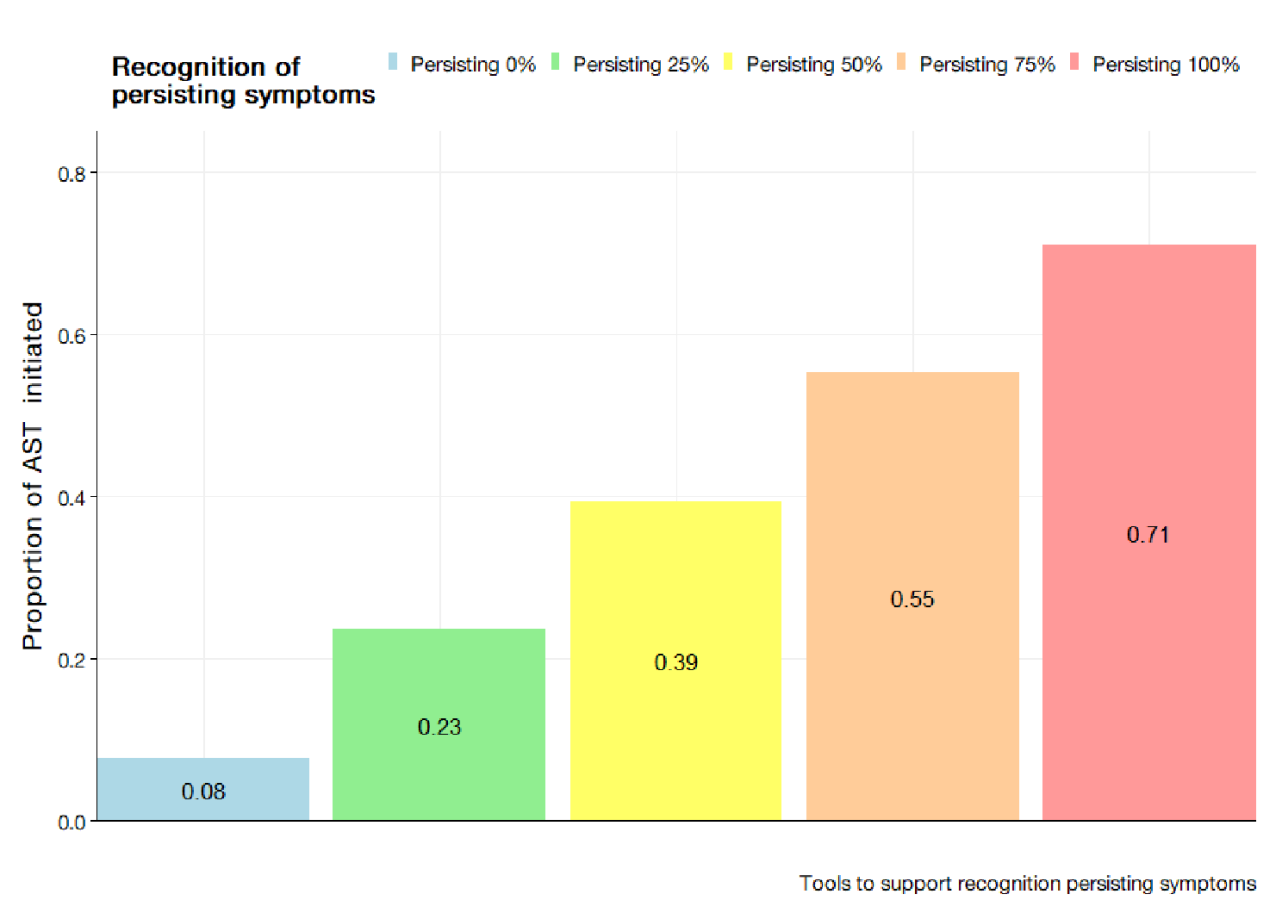
Proportion of AST initiation rates with scenario to introduce clinical support tools in assisting the recognition of persisting symptoms as produced by the BBN at values of 0, 0.25, 0.50, 0.75, and 1.00

The simulated results for the system-wide introduction of a clinical support tool to help clinicians decide whether to initiate an AST is presented in Fig. 7 for the SHSM and Fig. 8 for the BBN. In this scenario, a clinical tool is introduced to improving AMR testing. For the SHSM, this improves a clinician’s probability of testing, through the addition of another variable, that is modified by their adherence. In the BBN, the recognition of persisting symptoms should become more apparent and thus improve the probability of a test being initiated via modification of the persisting symptoms variable. The results indicate a general increase in proportion of tests initiated with a greater proportion of clinicians possessing such tool. At the lowest, 10–25% of clinicians having a clinical support tool would increase the proportion of tests from base scenario to 0.336 (95% CI; 0.320, 0.352). In the scenario where 76–100% of the clinician population have a tool, the model estimates a value 0.423 (95%CI; 0.398, 0.448) tests initiated. The presence of clinical tools which modify the recognition of persistence of symptoms was observed to greatly increase the probability of an test initiated with modification of the parameter to 100% yielding a 71% chance of AST test initiation.

#### Dual scenario improvements

**Fig. 9:**
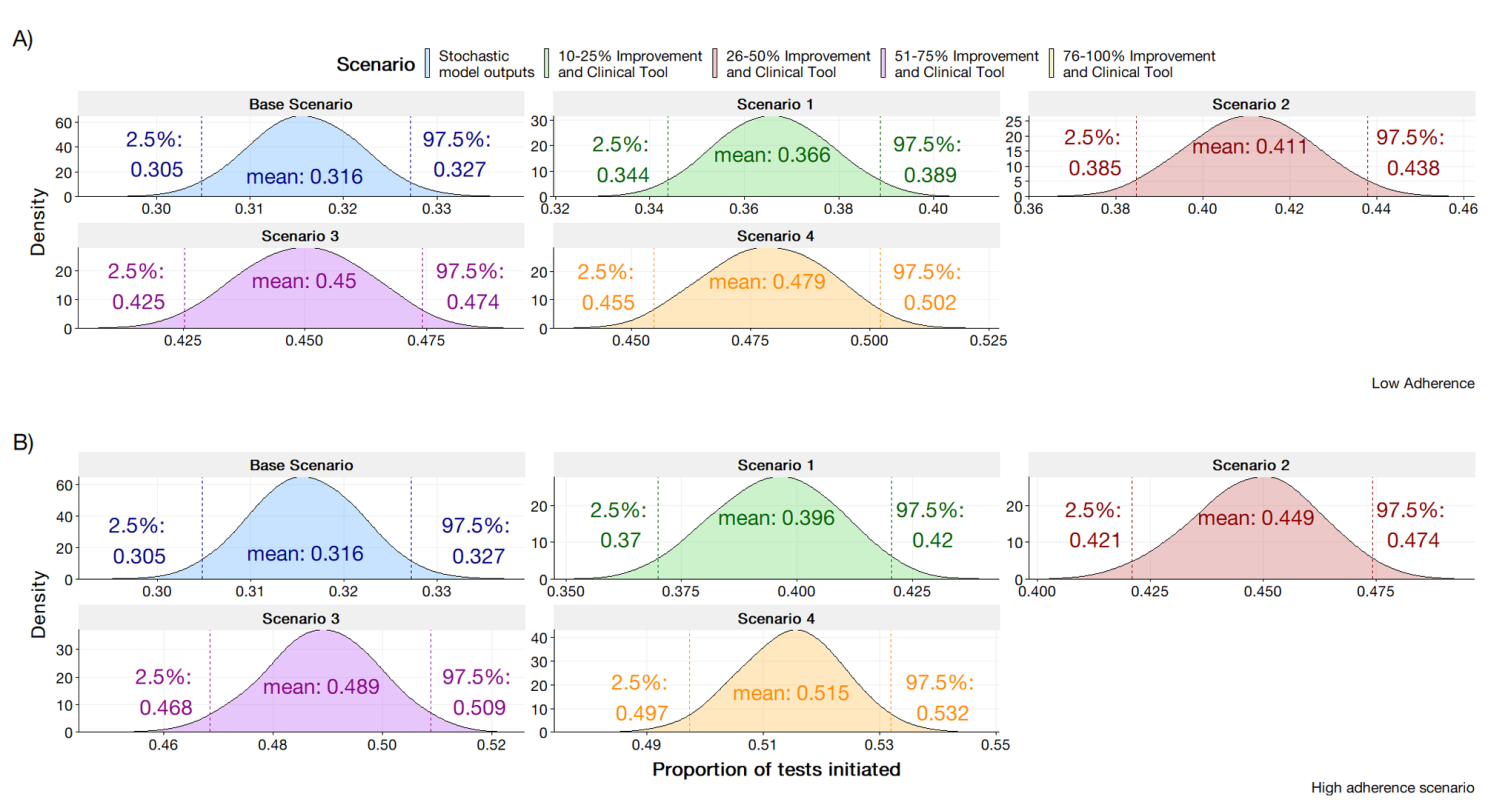
Stochastic health system’s model output based with clinical support tools and improvements in clinical awareness through stewardship or education. **(A)** Shows the proportion of tests initiated with a low adherence scenario with a 70/25/5 split in clinician population. **(B)** Depicts a high adherence scenario with a 5/90/5 split in clinician population

**Fig. 10:**
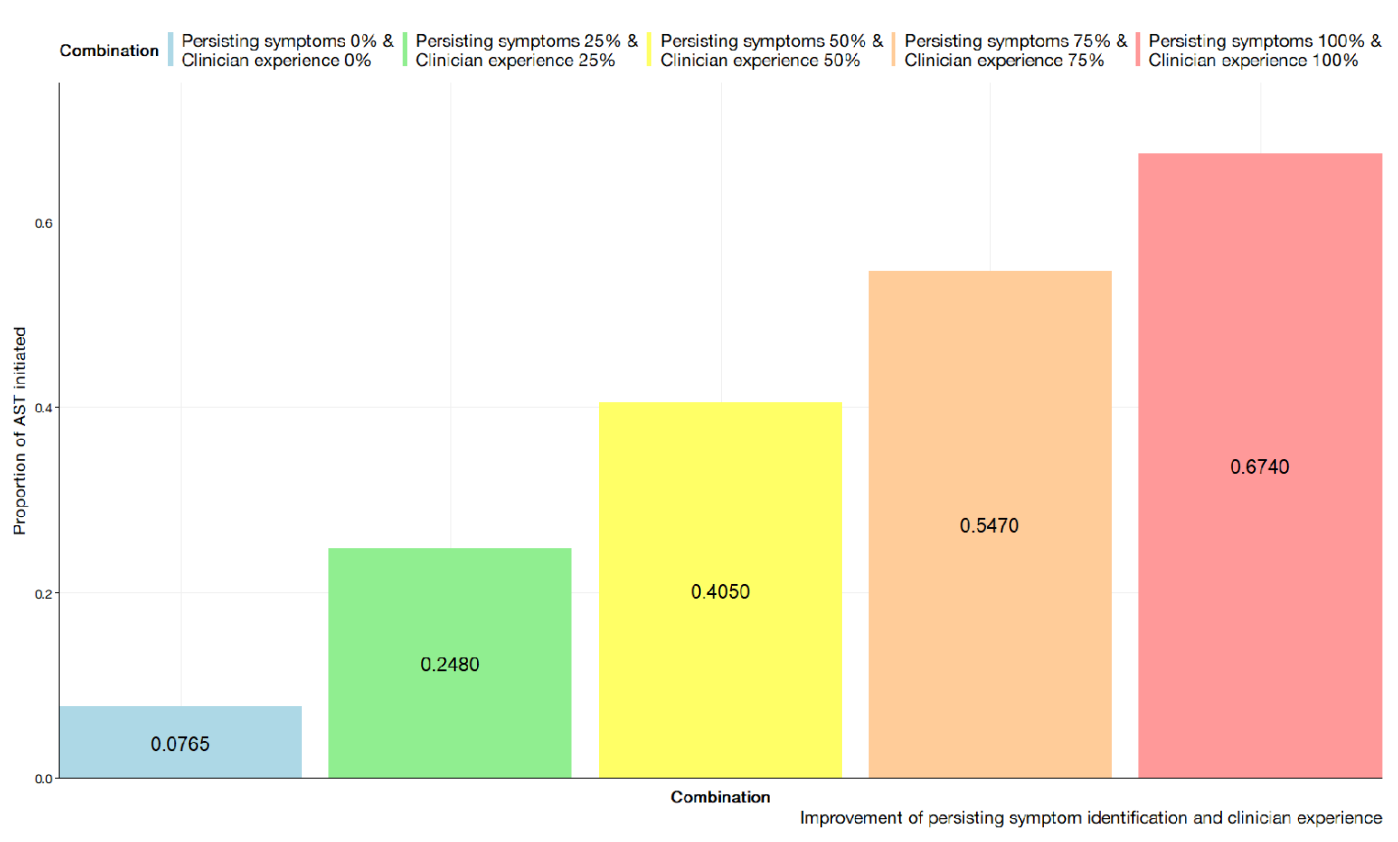
BBN based with clinical support tools and improvements in clinical awareness through stewardship or education with selected permutations

The results of introducing both clinical support and stewardship are presented below in Fig. 9 for the SHSM at base level adherence and Fig. 10 for the BBN. Generally, the SHSM notes improvements are noted at all levels of implementation. For the SHSM, improvements of 10–25% in clinical awareness and having clinical support tools, the model projects a statistically significant increase from the base scenario at 0.366 (95%CI; 0.344, 0.389) and 0.396 (95% CI; 0.370, 0.420) for the low and high adherence scenarios respectively. Increasing the experience and probability that symptoms have been persisting yielded a general increase with the BBN. By setting clinician experience to 100% and recognition of persisting symptoms to 100%, the probability for AN AST was observed to be 67.40%. For the SHSM, the greatest improvement is noted within scenario 4 at within the high adherence scenario with at 0.515 (95% CI; 0.497, 0.532). It is observed at greater levels of educational improvement and clinical support tool uptake, the proportion of tests initiated is projected to be greater when the clinician population is at higher adherence as opposed to lower. For incremental improvements at the levels 10–25% and 26–50% it has been the changes between high adherence and low adherence are not significant.

#### Improvements through mandates

**Fig. 11:**
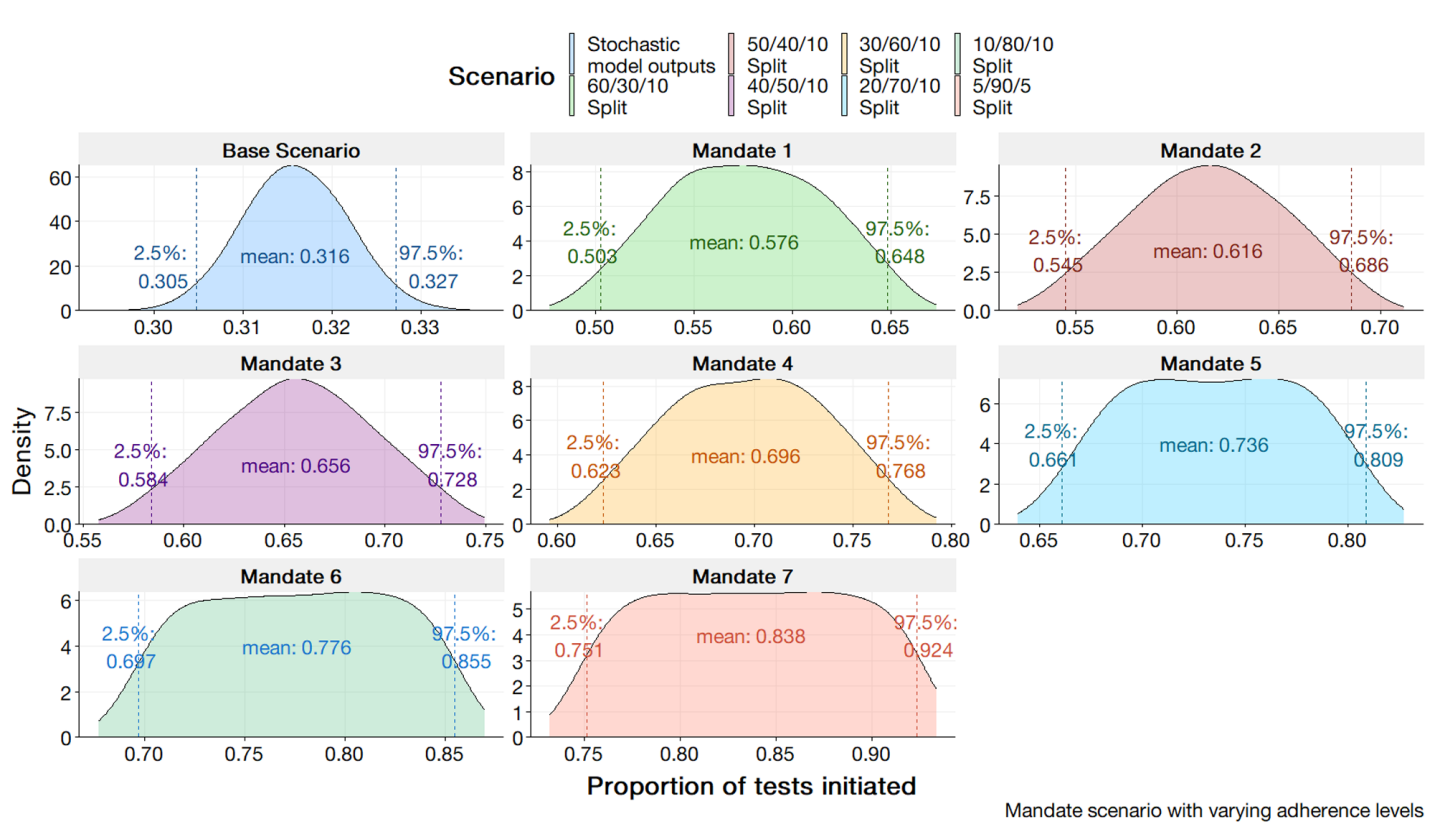
Stochastic health systems models output for proportion of AST tests initiated in the scenario of testing mandates. Clinician population is varied with differing average/high/low percentages

**Fig. 12:**
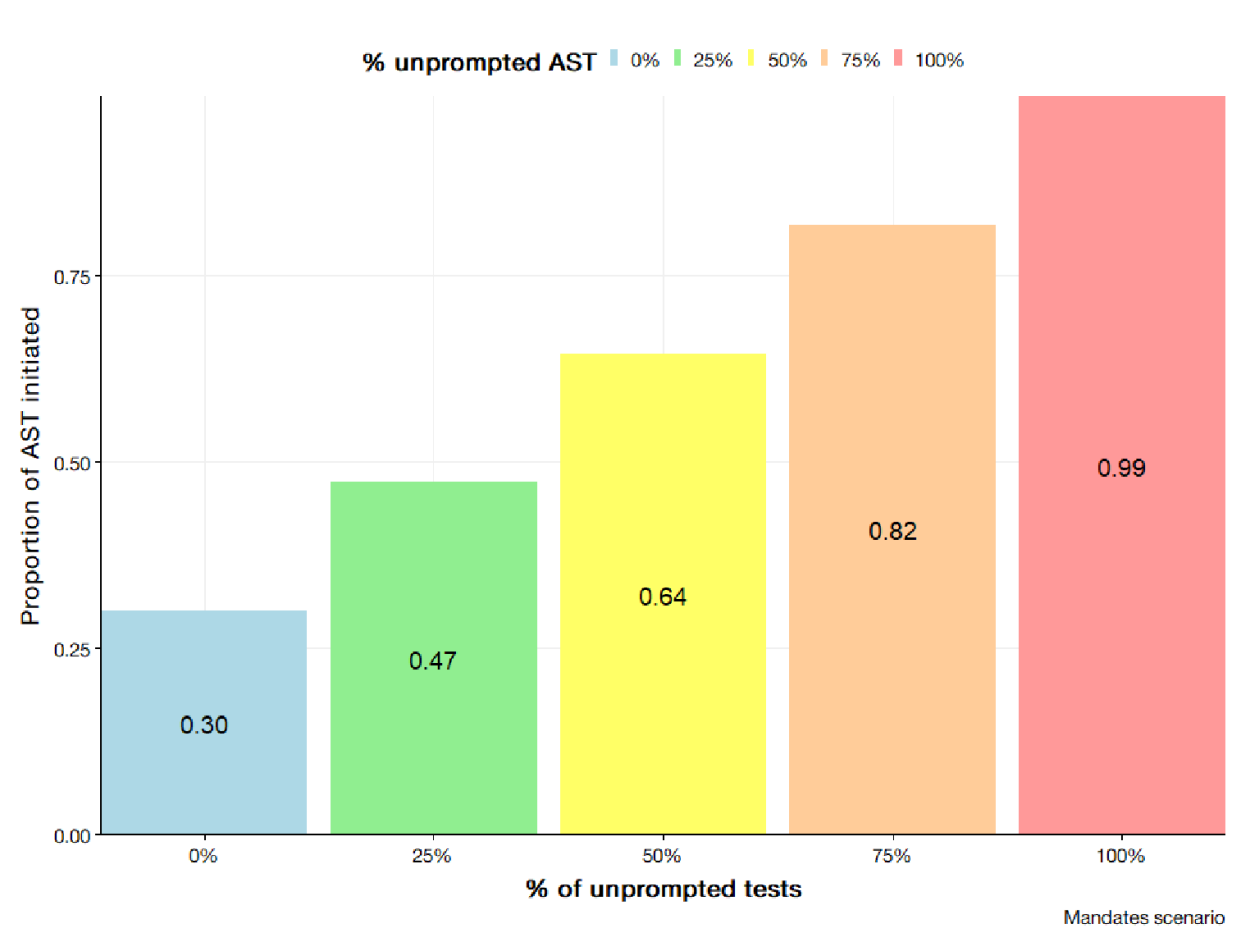
BBN outputs for AST initiation rates simulating a mandate scenario by modifying the unprompted AST parameter to values of 0, 0.25, 0.50, 0.75, 0.99

The introduction of mandates is assuming tests are initiated regardless of clinical decision making and deterministic factors. However, as tests are clinician initiated, estimates only depend on clinician adherence to guidelines. For the SHSM, tests are solely based on the adherence parameter regardless of the value of other parameters. In running the scenario for the BBN, the variable unprompted test has its values modified at increments. Results are depicted in Figs. 11 and 12 for the SHSM and BBN respectively. Generally, the trend noted by the results demonstrates a linear increase as the number of high adherence clinicians increases. At a conservative estimate of 60/30/10 (60% moderate adherence, 30% high adherence, 10% low adherence) the estimated proportion tested is 0.576 (95% CI; 0.503; 0.648). Optimistically with an estimated clinician population of 5/90/5, the estimated proportion of tests initiated is 0.837 (95% CI; 0.751, 0.924).

### Interventions to bayesian belief network

#### Scenarios with the bayesian belief network

The scenario analysis conducted on in the BBN for AST initiation offers comprehensive insights is presented in Table 1. The results demonstrate the influence of varying the parameters that are exclusive on the probability of initiating an AST.

The modification of epidemiological risk factors simulates a scenario whereby the population the clinician is in changes. Epidemiological risk factors showed a direct positive relationship. With no risk factors, the probability drops by 6.51% to 0.287 while increasing solely to high-risk populations yielded an increase of 16.161% to 0.358.

Adjustment of two or more parameters generally resulted in increases of AST initiation probability. Clinical experience was observed to dampen the gains in AST initiation probability when modified in permutations with others. The highest increase of 158.95% to a probability of 0.795 was observed when modifying persistence and epidemiological high-risk to 100% to simulate a high probability of persistence with an at-risk population. Moderate increases were seen with combinations of high risk and clinical experience (0.348) and persistence of symptoms with clinician experience (0.674). When all three parameters were increase together, AST initiation incrementally increased. The lowest probability was at 0.0064 in the absence of all parameters. A substantial increased was observed in the maximisation of all parameter values by 150.48% to 0.769.

**Table 1:** Scenario analysis using the Bayesian belief network for selected scenarios with probabilistic outputs of the AST test node and percentage of change from the initial state

Scenario^a^	New state probability of AST test	Percentage change from initial state^b^ (%)
**Epidemiological Factors (Proportion high risk)**		
High Risk 0%	0.287	−6.51
High Risk 25%	0.305	−0.65
High Risk 50%	0.322	4.88
High Risk 75%	0.340	10.75
High Risk 100%	0.358	16.61
**Persisting symptoms & Epidemiological Risk**		
Persistence 0% & High-Risk 0%	0.063	−79.19
Persistence 25% & High-Risk 25%	0.233	−24.10
Persistence 50% & High-Risk 50%	0.411	33.87
Persistence 75% & High-Risk 75%	0.598	94.78
Persistence 100% & High-Risk 100%	0.795	158.95
**Epidemiological Risk & Clinician Experience**		
High-risk 0% & Clinician experience 0%	0.320	4.06
High-risk 25% & Clinician experience 25%	0.324	5.24
High-risk 50% & Clinician experience 50%	0.330	6.97
High-risk 75% & Clinician experience 75%	0.338	9.17
High-risk 100% & Clinician experience 100%	0.348	11.78
**Epidemiological Risk, Clinician Experience & Persisting Symptoms**		
High-risk 0%, Clinician experience 0% & Persisting Symptoms 0%	0.064	−79.15
High-risk 25%, Clinician experience 25% & Persisting Symptoms 25%	0.246	−19.87
High-risk 50%, Clinician experience 50% & Persisting Symptoms 50%	0.422	37.459
High-risk 75%, Clinician experience 75% & Persisting Symptoms 75%	0.595	93.811
High-risk 100%, Clinician experience 100% & Persisting Symptoms 100%	0.769	150.48

^a^ Select scenarios are displayed with increments of 0.25 using Netica’s calibration function

^b^ Initial state of 0.307 on the AST initiated node

## Discussion

The primary aim of the study was to develop two models to identify the factors involved in the initiation of AST and identify the impact various systematic changes have on testing rates. To our knowledge, the paper presents two novel models developed to outline the causal pathways implicated in the initiation of AST. This is crucial as this step subsequently leads to the generation of a surveillance system data point.

The choice to model N. *gonorrhoea* AST initiation and simulate systematic changes was predicated on the necessity to understand the clinician-patient dynamics within AMR. This process is fundamental and a potentially rate-limiting step in the data generation processes implicated within AMR surveillance. Building a greater understanding of the underlying determinants would inform refinements of practices to improve testing and subsequently confidence in surveillance figures. The paper has done so by presenting two models. The decision to develop two distinct models was driven by the absence of literature defining the core characteristics of the clinician-patient dynamic within the context of AMR testing for N. *gonorrhoea*. Given the naïve state of empirical research, the dual-model approach was deemed necessary to hypothesise foundations for the resultant model structures.

Interestingly, the contrast presented by the SHSM and BBN offers collective value in understanding the intricate dynamics of clinician decision making in the AMR context. The models encapsulate the stochastic and deterministic elements of clinical behaviour and offer frameworks that bridge gaps within existing AMR literature. Evidently, there is a difference in conceptualisation of how clinical experience works. The BBN would suggest more experienced clinicians are less likely to initiate an AST which contrasts the SHSM’s randomness assumption. The presumption for the behaviour is predicated on literature suggesting experienced clinicians can better tolerate uncertainty and thus led to lower diagnostic testing [30]. Despite this fundamental difference, the results of the scenario analysis are identical in general trend. Indeed, the only underlying difference is the magnitude of the intervention effect on AST initiation rates. Given further advancements in literature which highlight the nuances of AMR test behaviour, a clear choice for the models and subsequent paradigm can be chosen.

Despite the foundational differences, both models converge on outcomes as demonstrated within the scenario analysis. The convergence suggests while the approach to modelling clinician behaviour can vary, the overarching influences on AST initiation – like those of clinician experience and adherence to guidelines – remain constant across the stipulated conceptual frameworks. The contrast and complementarity of the two models suggest the choice between the two models should be guided by the characteristic nature of the population. Indeed, the SHSM is suited to environments where clinician behaviour may be highly variable and influenced by unpredictable factors that not been explicitly outlined. Conversely, the BBN’s deterministic nature would be ideal for settings where data on causal relationships is robust and well-documented. Overall, the choice of the model for best prediction within the Australian context would require a better understanding of the AMR testing dynamics present within clinical practice.

### Strengths and limitations of the models

There are inherent strengths and limitations that must be acknowledged in the interpretation of the model outputs.

In the interpretation of the model outputs, a key characteristic of the models that must be considered is the validity of the models. One strength presented by the paper for the models is their internal validity. The internal validity of the constructed models is supported by their foundations in existing STI literature and their validation to empirical historical data. For the stochastic health systems model, validity has been sought through the parameterisation of historical data. The model’s alignment with the parameterised historical data would indicate a credible simulation of health system’s level AST initiation dynamics. For the BBN, a mixture of empirical epidemiological data and expert opinion have been used to refine the system structure and elucidate the causal pathways influencing AST initiation. The sensitivity analysis conducted further validates the structural and parametric integrity by reflecting sound relational integration between nodes. Moreover, with the BBN is robust with high predictive capability.

An interesting point of discussion arises when examining the external validity. A potential limitation presented is the absence of external validation due to the novelty of the models. Though the developed models exhibit extensive internal validity with alignment to literature and expert opinion, critical appraisal of the models reveals gaps in literature that, if addressed, could further refine the generalisability of the model outputs. For instance, the causal relationships and determinants for a clinician to initiate an AST have no overwhelming consensus within the surrounding literature. The model building methodology has sought to address this gap by postulating two contrasting paradigms whereby the process could either be random yet deterministic at a health systems level or completely deterministic at a clinical level. This bifurcation of the paradigms serves purposes beyond theoretical considerations as it forms the basis on how the system could be examined. Further research into consolidating a consensus in the processes implicated in test generation would provide further commentary regarding external validity of the models.

The model building methodology presented within this paper has limitations that are essential to be acknowledged. The SHSM and BBN have pragmatic underlying assumptions that have been implemented to achieve parsimony. The model’s presuppose uniform access to diagnostic care, consistent resistance patterns, uniform diagnostic practices and sufficiency of historical data to for validation. These assumptions present a layer of abstraction to the clinician-patient dynamic. However, the assumptions are a necessity in model building to (1) simplify model interpretation and (2) absence of literature to quantitatively define the influence these assumptions have on outputs. Therefore, it is imperative for model outputs to be viewed as approximations. Furthermore, parameterisation is challenging in the absence of rich literature to delineate the influence variables exert upon one another. The stochastic model’s strength is the capacity to circumvent this via utilising AMR data associating population-level factors with outcomes. The BBN faces more pronounced challenges due to the reliance on definitive causal pathways and the population of CPTs. Expert opinion has been sought for CPT population and outputs are within values given by historical data. Overall, there has been methodological conveniences implemented, necessitated by current limits in the granularity of AMR patient-clinician dynamics that could be further improved with further research to understand and expound on the intricacies presented within the models.

### Scenario analyses

The SHSM and BBN models and their respective simulation of scenarios highlight potential system level improvements to leverage in the endeavour to increase AST proportions.

Interestingly, the SHSM’s projections suggest improvements in clinician awareness through stewardship could significantly increase AST initiation rates to mirror the early-stage proportions of isolates tested recorded by the AGSP. The increase in clinician awareness by 76–100% is indicative of a return to historical test data proportions. The increase from 0.316 to 0.410 aligns with the reported value in 2008 [21]. Further extrapolating trends would delineate a relationship with awareness and proportions tested. Indeed, pre-2008 AGSP data indicates proportions greater than 40% [21] and this would mirror projections made by modifying clinician awareness beyond the stipulated 76–100% presented in the results. The parallel drawn would suggest a previous heightened awareness of the AGSP and AMR which has subsequently waned over time with determinants, such as the uptake of NAAT [8], leading to a reduction in current testing rates. The SHSM’s findings provide a foundation for further action to be taken to revitalise educational efforts regarding AMR within the clinical space.

The SHSM model’s outputs highlights deployment of clinical support tools as a pragmatic scenario for improving the AST initiation rates. Surrounding literature regarding adoption of support tools has consolidated the consensus regarding uptake of the tools in clinical practice [31–33]. The results of the model would indicate similar level of improvements to that of modifying clinician knowledge parameters with 0.396 (95%CI; 0.373, 0.420) and 0.404(95% CI; 0.383, 0.426) respectively. The similarity would suggest that clinical support tools for AMR could serve as an adjunct or alternative to direct educational interventions which is problematic due to quantification of AMR knowledge. While promising, it is imperative to consider real-world factors such as accessibility and acceptability which are not captured by the model [34]. Further contextualised assessment of the criteria is needed to realise the potential gains highlighted by the model in future research.

Adherence to guidelines emerge as a substantiative determinant in understanding and improving AST initiation rates as indicated by the SHSM dual strategy scenario. The model is fundamentally predicated conversative estimation of clinician adherence to guidelines which has been rationalised by surrounding literature concerning clinical practice literature [35]. Improvements strategies that concurrently bolster clinical knowledge and integrate clinical support tools could substantially be elevated with a health workforce with higher adherence tendencies.

The scenarios presented by the BBN present an interesting paradigm regarding improvement in AST initiation being largely influenced by patient level factors. Within the scenario analyses of the BBN, substantial increases were noted when patient level factors were modified. More notably, increasing the probability that has persistence of symptoms can yield two-fold increases in probability for a test to be initiated. Assuming uniformity in testing probability across a population, it would suggest more tests would be initiated if a larger proportion of cases originate from either a higher risk group or greater amounts of individuals having persisting symptoms. This is possible with increased patient awareness of AMR. Though, literature has not substantiated a large research corpus surrounding public awareness on AMR and impact on patients initiating diagnostic testing processes. However, for surveillance purposes, it is likely such scenario would lead to increased bias within sampling which limits the representativeness of the system.

### Case for mandated susceptibility testing

Both models would indicate the greatest improvement in AST initiation is observed with testing mandates. As observed in the probabilistic outputs unprompted tests and mandates for the SHSM, rates greater than 90% can be achieved. The lower value output by the SHSM considers potential stochasticity at a systems level whereby adherence to guidelines is an influential determinant. Indeed, the mandate scenario has legislative grounds for plausibility in Australia with current guidelines indicating mandatory notification of N. *gonorrhoea* infections. In such case of mandates, every N. *gonorrhoea* notification would have a parallel culture initiated or the predominant diagnostic method includes sensitivity testing. Diagnostic AMR literature has identified methods for rapid ASTs which have the potential for integration into existing diagnostic frameworks [36]. To bolster the confidence in the surveillance systems, the mandate of AST tests presents as a potential mechanism.

There are concerns regarding the practicality of mandates given the potential stress on laboratory capacity given the rapid increase of N. *gonorrhoea* notifications within Australia. The feasibility and necessity of achieving absolute certainty within surveillance system is debatable. The paradigm presented is idealistic to the designated objectives of the system to monitor and evaluate trends. Without further assessment of capacity, the materialisation of such paradigm is unlikely to occur. Pragmatic approaches employing survey design principles and leveraging systematic randomised testing could enhance the representativeness of the surveillance system while mitigating potential laboratory strain. For example, the instigation of biweekly AST testing on all notifications could result in a testing proportion of 0.50. Furthermore, whilst AST initiation rates may exceed 90%, as indicated by both model’s mandate scenarios, there needs to be the considerable of culture failure. Mohammed, et al. [37] in an analysis of N. *gonorrhoeae* isolates found that despite isolates being available for culture almost half of these failed and were not reattempted. At the current state, further inference on N. *gonorrhoea* AMR epidemiology based off any gains from AST initiation, requires the consideration culture failure rate in lieu of more robust methods.

## Conclusions

This study quantified the determinants influencing AST initiation for N. *gonorrhoea* within the Australian context and assessed the impact simulated, systematic changes on the system would have on test rates to enhance the AGSP. Two models were employed to achieve the objectives with a mathematical model, SHSM, and BBN used to represent different paradigms in how to model the intricacies of the patient-clinician dynamic. Clinician education, support tools, and adherence to guidelines were identified as potential leverages for further policy interventions to increase AST initiation. For the BBN, changes in population characteristics were identified to be most influential in clinical decision-making regarding AST initiation. Findings suggest underlying trends in wanning engagement in AMR awareness with potential interventions targeted, either through tools or clinical education interventions as mechanisms for rectification. Both models support legislative changes regarding AMR testing frameworks through encompassing legislation. Though practicality issues may arise with laboratory capacity constraints, pragmatic solutions with randomised testing strategies offer a mechanism to mitigate strain. Overall, the study offers insights on how to improve AMR surveillance for N. *gonorrhoea* within Australia. However, the study suggests there are still innate gaps to the understanding of the AMR patient-clinician dynamic that has not yet been thoroughly elaborated upon to achieve granularity. This is an area necessitates further investigation by future studies.

### Electronic supplementary material

Below is the link to the electronic supplementary material.

Supplementary Material 1

Supplementary Material 2

Supplementary Material 3

## Data Availability

All data is included within the supplementary files.
