# Supplementary material for: Policy, practice, and prediction: model-based approaches to evaluating N. gonorrhoeae antibiotic susceptibility test uptake in Australia
Source: BMC Infect Dis. 2024 May 17;24:498. doi: 10.1186/s12879-024-09393-y (PMC11100046; doi:10.1186/s12879-024-09393-y)
Supplement: Supplementary file 1 — Supplementary Material 1 [file 12879_2024_9393_MOESM1_ESM.pdf]

Supplementary File 1 Combined Research

Systematic Search

To identify the factors, a systematic search has been completed to review literature regarding sexually transmitted infection (STI) testing. This scope has been chosen as the literature regarding AMR testing is currently naïve and there is insufficient literature to inform decision making. However, with thematic similarities present (N. *gonorrhoeae* as an STI), it is plausible for the same factors to be present in N. *gonorrhoeae* testing as found with STI testing. The following strategy for informing the model building process is noted in Supplementary Table 1.

Supplementary Table 1 Systematic search protocol for model building

Step	Detail
**Database Selection**	PubMed, Scopus, Web of Science
**Search Terms**	"STI testing", "barriers to STI testing", "facilitators of STI testing", "STI point-of-care testing", "healthcare provider STI management", "patient attitudes towards STI testing", "STI testing policies"
**Search Queries**	("sexually transmitted infections" OR "STI") AND ("testing" OR "screening") AND ("barriers" OR "challenges" OR "obstacles") AND ("facilitators" OR "enablers" OR "drivers") AND ("healthcare providers" OR "clinicians" OR "physicians" OR "nurses") AND ("patient attitudes" OR "patient perceptions") AND ("point-of-care" OR "rapid tests") AND ("policy" OR "guidelines")
**Inclusion Criteria**	Studies that focus on STI testing practices, barriers, and facilitators; published in the last 10 years; peer-reviewed articles; available in English.
**Exclusion Criteria**	Studies not related to STI testing; older than 10 years; not peer-reviewed; not available in full text; non-English articles.
**Screening Process**	Two reviewers independently screen titles and abstracts for relevance; disagreements resolved by discussion or third reviewer.
**Eligibility and Full-text Review**	Full-text articles assessed for eligibility; data extraction for relevant articles including study design, population, outcomes, and findings related to STI testing.

**Search Strategy Documentation**	Record number of articles identified, screened, eligible, and included document reasons for exclusion at full-text stage; Further hand search of bibliography for any more relevant studies was undertaken.
**Search Strategy Adaptation**	If initial search yields too many irrelevant results, refine search terms; consult subject matter experts for additional or alternative keywords.
**Implications for model building**	Implications of this will inform model parameters that need to be included via extraction into a table and the formation of a influence matrix.

Translation

An influence matrix table will be created to inform the model building process.

Supplementary Table 2 Research literature search results with the identification of factors with sources barriers and facilitators for STI testing.

Title	Article Type	Main points	Factors	Source
**Uptake of and factors associated with testing for sexually transmitted infections in community-based settings among youth in Zimbabwe: a mixed-methods study.**	Research Article	• Study nested within CHIEDZA trial.<br>• **Individuals offered non-selectively confidential testing for chlamydia and gonorrhoea** (regardless of symptoms)<br>• Authors used questionnaire to record socio-demographic information.<br>• Separate interviews for in-depth information.<br>• Analysis conducted through logistic regression.<br>• Symptom and perceived risk of STI's increased uptake of testing<br>• Misinformation, stigma, and marital status negatively affected testing.<br>• Single/boyfriend/girlfriend decreased uptake, whilst being married increased.<br>• Employment was negatively associated with test uptake.<br>• Concerns with confidentiality	**Patient Factors**<br>• **Symptoms** [*Facilitator*]<br>• **Perceived Risk** *[Facilitator]*<br>• **Misinformation** *[Barrier]*<br>• **Stigma** *[Barrier]*<br>• **Marital status** [Facilitator/Barrier]<br>• **Employment** [Facilitator/Barrier]<br>• **Stigma** [Barrier]<br>• **Confidentiality** [Barrier]	[1]
**Facilitators and barriers to point-of-care testing for sexually transmitted infections in low- and middle-income countries: a scoping review.**	Scoping Review	• STI management in LMIC is symptomatically managed.<br>• **Point of care tests (POCTs)** can increase better understanding of prevalence of STI.<br>• 82 articles included.<br>• 7 overarching themes related in implementation of POCTs in LMIC<br>• 1. Ideal test characteristics<br>• 2. Client factors	**Patient Factors**<br>• Acceptability [Facilitator]<br>• Feasibility [Facilitator]<br>• Accuracy [Facilitator]<br>• Rapid/Timeliness [Facilitator]<br>• Cost of attendance [Barrier]<br>• Cost of treatment [Barrier]<br>• Perceived risk [Facilitator]<br>• Misinformation [Barrier]	[2]

		• 3. Healthcare provision factors<br>• 4. Policy, infrastructure, and health system factors<br>• 5. Training, audit, and feedback.<br>• 6. Reaching new testing environments<br>• 7. Dual testing	• Trust [Barrier]<br>• Stigma [Barrier]<br>• Individual preferences [Barrier]<br>• Socio-demographics [Barrier/Facilitator]<br>• Environment [Barrier/Facilitator]<br>• Home-testing [Facilitator]<br>• Rurality/remoteness [Barrier]<br>**Clinician Factors**<br>• Accuracy [Facilitator]<br>• Confidence [Barrier/Facilitator]<br>• Equipment-free [Facilitator]<br>• Rapid/Timeliness [Facilitator]<br>• Reliability (adverse conditions) [Facilitator]<br>• User-friendliness [Facilitator]<br>• Healthcare flow (Consultation time, waiting times, clinic flow) [Barrier/Facilitator]<br>• Staff knowledge/motivation [Barrier/Facilitator]<br>• Documentation [Barrier]<br>• Authority [Barrier]<br>• Supply [Barrier]<br>• Funding [Barrier]<br>• Engagement with stakeholders [Facilitator]<br>• Integration of learning [Facilitator]<br>• Guidelines [Facilitator]<br>• Health systems [Barrier/Facilitator]<br>• Training [Barrier/Facilitator]<br>• Supervision/remedial training [Facilitator]	

			• Quality assurance [Barrier]<br>• Environment [Barrier/Facilitator]<br>• Remoteness [Barrier<br>• Dual testing [Facilitator]<br>• Multiple testing [Facilitator]	
**Healthcare provider perspectives on managing sexually transmitted infections in HIV care settings in Kenya: A qualitative thematic analysis.**	Research Article	• STI burden has increased in Kenya.<br>• Objectives to describe **healthcare provider (HCP)** knowledge and practices, attitudes, and beliefs, and identify structural and environmental factors affecting STI management.<br>• In-depth interviews obtained with 87 HCP working in 21 high volume comprehensive HIV care centres (CCCs).<br>• Varied staff mix (e.g., Doctors, Nurses, Pharmacists)<br>• Identified were:<br>• 1. Knowledge and practices<br>• 2. Beliefs and attitudes<br>• 3. Structural and environmental factors affecting STI management	**Clinician Factors**<br>• Knowledge [Facilitator]<br>• Screening practices [Barrier/Facilitator]<br>• Priority [Barrier]<br>• Reporting practices [Barrier/Facilitator]<br>• Supervision/Training [Barrier]<br>• Service integration [Facilitator/Barrier]<br>• Prior beliefs [Facilitator/Barrier]<br>• Stigma [Barrier]<br>• Awareness of policy [Barrier/Facilitator]	[3]
**Healthcare Provider Perceptions of a Sexually Transmitted Infection Self-Testing Program in an HIV Care Clinic**	Research Article	• Evaluation of STI self-testing program in HIV clinic in Seattle, Washington.<br>• Self-testing process including labelling collection kits, urine sample, and specimens.<br>• Tests initiated by clinicians (91% referred MSM to self-testing).<br>• Mixed method programs evaluation assessing healthcare provider acceptability through interviews and focus group.	**Clinician Factors**<br>• Timeliness [Barrier/Facilitator]<br>• Patient acceptability [Barrier/Facilitator]<br>• Patient accessibility [Barrier/Facilitator]<br>• Unclear direction of patients [Barrier]<br>• Incorrect sample collection and labelling [Barrier]<br>• Patient capability to conduct test and timeliness [Barrier]	[4]

**Survey of Health Care Providers' Practices and Opinions Regarding Bacterial Sexually Transmitted Infection Testing Among Gay, Bisexual, and Other Men Who Have Sex with Men**	Research Article	• Study aimed to assess bacterial STI testing practices amongst providers for gay, bisexual, and other men who have sex with men (GBMSM) in Toronto, Canada.<br>• Survey to collect data from 95 healthcare providers (55% response rate)<br>• Respondents: (70% physicians) and (65% primary care)	**Clinician Factors**<br>• Insufficient consultation time [Barrier]<br>• Difficulty raising STI testing in unrelated consultations [Barrier]<br>• Capacity [Barrier/Facilitator]<br>• Resources [Barrier]<br>• Clearer guidelines [Barrier/Facilitator]<br>• Timeliness (Express Testing) [Facilitator]<br>• Patient collected specimens [Facilitator]<br>• Provider alerts [Facilitator]<br>• Practice volume [Barrier/Facilitator]<br>**Patient Factors**<br>• Acceptability [Barrier]<br>• Cultural competencies (Stigma) [Barrier]	[5]
**Improving care for sexual transmitted infections**	Comment ary	• Rising rates of STIs require a public health response.<br>• STI clinics have played a central role in providing STI care.<br>• Different settings have different strengths.<br>• Primary – focus on improving STI screening rates, expedited partner therapy.<br>• Family planning – opportunities to serve partners with STI services.<br>• STI clinics – consultation and referral evolve from safety net	**Clinician Factors**<br>• Expertise in STI [Barrier/Facilitator]<br>• Clinic volume [Barrier/Facilitator]<br>• Education [Facilitator]<br>• Appropriate staffing [Barrier/Facilitator]<br>• Standards of care [Barrier]<br>**Patient Factors**<br>• System accessibility [Barrier/Facilitator]	[6]
**Sexually Transmitted Disease Testing Protocols, Sexually Transmitted**	Cross-sectional study	• Aim of the study was to assess the frequency and type of STD testing and sexual risk assessment	**Clinician Factors**<br>• Electronic submission [Facilitator]<br>• Guidelines [Facilitator]	[7]

**Disease Testing, and Discussion of Sexual Behaviors in HIV Clinics in Los Angeles County**		practices among HIV care providers in Los Angeles County.<br>• 36 medical directors, HIV clinic care coordinators, or practicing HIV providers. 184 clinicians involved.<br>• 50% of clinics reported electronic protocol for STD		
**Opportunities and barriers to STI testing in community health centres in China: a nationwide survey**	Cross-sectional survey	• Paper aimed to investigate the experiences and attitudes of primary care practitioners (PCPs) in China regarding their role in STI testing and management at the primary care level to identify barriers and solutions for implementing STI programmes.<br>• PCPs in are keen to be trained and upskilled.<br>• Logistical, attitudinal, and educational barriers to overcome effective implementation of STI programmes.<br>• Barriers addressed could lead to increased testing.	**Clinician Factors**<br>• Training [Barrier/Facilitator]<br>• Perceived non-benefit [Barriers]<br>• Stigma (Driving other patients away) [Barrier]<br>• Difficulty [Barrier]<br>• Clinician safety [Barrier]<br>• Time [Barrier]<br>• Resources [Barrier]<br>• Concerns patient compliance [Barrier/Facilitator]<br>**Patient Factors**<br>• Accessibility [Barrier]<br>• Stigma [Barrier]	[8]
**The Role of Provider Interactions on Comprehensive Sexual Healthcare Among Young Men Who Have Sex With Men**	Research Article	• The study aimed to examine differences in HIV testing categories by demographic/psychosocial variables among young men who have sex with men (YMSM).<br>• Online survey with 1,528 YMSM<br>• Younger participants more likely to be seen by medical provider than older counterparts.<br>• Participants identifying as gay/bisexual more likely to be tested for HIV.<br>•	**Patient Factors**<br>• Age [Barrier/Facilitator]<br>• Sexuality [Barrier/Facilitator]<br>• Insurance/financial [Barrier/Facilitator]<br>• Comfort [Facilitator]	[9]
**Do healthcare professionals and young adults about the National Chlamydia**	Cross-sectional survey	• Article aim was to investigate the extent to which healthcare professionals (HCPs) and young people	**Clinician Factors**<br>• Awareness [Barrier]<br>• Adherence [Barrier]	[10]

**Screening Programme? Findings from two cross-sectional surveys.**		(YP) are aware of, and adhere to, the National Chlamydia Screening Programme (NCSP).<br>• One survey conducted among genitourinary medicine (GUM) and non-GUM professionals.<br>• One survey conducted among young people attending a GUM clinic in England<br>• Surveys found variable awareness of NCSP guidance<br>• Room to improve NCSP awareness among HCP and YP	**Patient Factors**<br>• Awareness [Barrier]<br>• Partner knowledge [Facilitator]	
**How do general practitioners test and treat gonococcal infections in the Australian Capital Territory? Implications for disease surveillance and control**	Research Article	• The aim of the article was to explore conformity of self-reported clinical practice with sexually transmitted infection guidelines in general practice, ACT Health.<br>• Mixed methods study with a quantitative analysis of a written survey and grounded theory-based<br>• 23 GPs and 1 NP completed the survey.<br>• Key concepts were coded where labels were manually assigned to words and phrases to represent ideas.	**Clinician Factors**<br>• Limited time [Barrier]<br>• Limited clinician understanding of associated benefits [Barrier]<br>• Uncertainty about approaching sexual health [Barrier]<br>• Patient preferences [Barrier]<br>• Clinician attitudes [Barrier]	[11]
**Clinician Adherence to Recommendations for Screening of Adolescents for Sexual Activity and Sexually Transmitted Infection/HIV**	Retrospective cross-sectional study	• Study aimed to measure frequency of sexual history and screening documentation for STI and HIV by clinicians during adolescent well visits across primary care practices.<br>• 29 CHOP owned primary care centres<br>• Infrequent documentation of sexual histories and STI/HIV screening done on adolescent patients<br>• Primary outcomes were frequency of STI/HIV screening and performance of GC/CT and HIV testing.<br>• 20% of patients sexually active received recommended screening	**Clinician Factors**<br>• Patient factors [Barrier/Facilitator]<br>• Clinician gender [Barrier/Facilitator]<br>**Patient Factors**<br>• Age [Barrier/Facilitator]<br>• Ethnicity [Barrier/Facilitator]<br>• Insurance [Barrier/Facilitator]	[12]

		• Patient factors implicated in documentation and STI/HIV screening<br>• Female clinicians more likely to conduct sexual risk assessments		
**What Qualities Are Most Important to Making a Point of Care Test Desirable for Clinicians and Other Offering Sexually Transmitted Infection Testing?**	Research Article	• The aim was to identify the ideal point-of-care test for sexually transmitted infections based on the preferences.<br>• Conducted among STI professionals and experts.<br>• High sensitivity, specificity, timeliness, and low cost were identified to be important for STI tests<br>• Reimbursement more prevalent in the US as a contributing factor<br>•	**Clinician Factors**<br>• Low cost [Facilitator]<br>• Reliability of test [Facilitator]<br>• Trade-off [Barrier]	[13]
**Factors associated with a clinician's offer of screening HIV positive patients for sexually transmitted infections, including syphilis.**	Research Article	• Study aimed to assess whether Quality Improvement Scotland for sexual health offered to HIV positive individuals are being met by genitourinary (GU) medicine clinic for screening of STI.<br>• 509 patients who visited GU medicine clinic.<br>• 64% of patients documented offer an STI screen within 1 year of recent visit, 66.6% accepted the offer.<br>• Logistic regression used to identify predictors for testing.	**Clinician Factors**<br>• Authority [Barrier/Facilitator]<br>**Patient Factors**<br>• Sexual orientation [Facilitator]<br>• Relationship status [Barrier/Facilitator]<br>• Receipt of antiretrovirals/syphilis testing [Facilitator]<br>• Gender [Facilitator]<br>• CD4 levels (biomarker) [Facilitator]	[14]
**Clinical Factors Associated With Accurate Presumptive Treatment of Neisseria gonorrhoeae Infections in Men Who Have Sex with Men and Transgender Women**	Cross-sectional study	• Aim of the study was to identify clinical factors associated with accurate treatment of Neisseria *gonorrhoeae* infections in men who have sex with men (MSM) and transgender women.<br>• Study done across patients attending between 2016 and 2019 in California and Florida.<br>• Multi-variate logistic regression analyses conducted from 2013 – 2017 with 42,050 patient encounters.	**Clinical Factors**<br>• Authority [Barrier/Facilitator]<br>• Expertise [Barrier/Facilitator]<br>**Patient factors**<br>• Discharge, dysuria, rectal discharge. [Facilitator]<br>• High risk sexual behaviour [Facilitator]<br>• Race [Facilitator]	[15]

		• Age, race, gender, HIV status, and region were considerations in the model.<br>• Important factors identified were urethral discharge, dysuria, rectal discharge, high risk sexual behaviour and history.	• Gender [Facilitator]<br>• Age [Facilitator]<br>• HIV status [Facilitator]<br>• Region [Facilitator]<br>• History [Facilitator]	
**Why are men less tested for sexually transmitted infections in remote Australian Indigenous communities? A mixed-methods study**	Research Article	• The article aims to investigate why men are less tested for sexually transmitted infections in remote Australian Indigenous communities.<br>• Study in Northern Territory of Australia<br>• Health services generally provided by one community clinic.<br>• Audit conducted where a known disparity has persisted.<br>• Young men expressed a preference for male centred spaces and clinicians.	**Clinician Factors**<br>• Responsiveness [Barrier]<br>• Ethnicity [Barrier]<br>• Sex [Barrier/Facilitator]<br>**Patient Factors**<br>• Stigma [Barrier]	[16]
**Narrative review of the barriers and facilitators to chlamydia testing in general practice**	Narrative Review	• The aim of was to identify barriers and facilitators to chlamydia testing in the primary care setting in Australia.<br>• Three levels of barriers identified in patient, general practitioner, and general practice.	**Clinician Factors**<br>• Knowledge [Barrier/Facilitator]<br>• Awareness [Barrier/Facilitator]<br>• Training [Barrier/Facilitator]<br>• Workload and time [Barrier/Facilitator]<br>• Social context [Barrier/Facilitator]<br>• Education [Facilitator]<br>• Adequate resources and time [Facilitator]<br>**Patient Factors**<br>• Stigma [Barrier]<br>• Confidentiality [Barrier]<br>• Inadequate promotional materials [Barrier]<br>• Accessibility [Barrier]<br>• Age [Barrier/Facilitator]	[17]

			• Comfort [Barrier/Facilitator]<br>• Knowledge [Barrier/Facilitator]<br>• Awareness [Barrier/Faciliator]	

Supplementary Table 3 Influence matrix with facilitators (+), barriers (-), both (+/-) for clinicians, patients, and systematic factors

Factor	Type	Influence	References
Symptoms/Perceived Risk	Patient	+	[1]
Marital Status	Patient	+/-	[1]
Employment	Patient	+/-	[1]
Misinformation	Patient	-	[1], [2], [8], [17]
Stigma	Both	-	[1], [2], [3], [4], [5], [8], [16], [17]
Confidentiality Concerns	Patient	-	[1]
Acceptability/Feasibility	Both	+	[2], [4], [5], [9], [12]
Cost Barriers (Testing & Treatment)	Patient	-	[2]
Home-testing	Patient	+	[2], [4]
Rurality/Remoteness	Patient	-	[2], [16]
Knowledge & Training	Clinician	+	[3], [4], [8], [13], [17]
Structural/Environmental Factors	Both	+/-	[3], [4], [14]
Supervision/Training	Clinician	+/-	[3], [14]
Ideal Test Characteristics	Clinician	+	[2], [13]
Healthcare Provision Factors	Clinician	+/-	[2], [3]
Policy/Infrastructure	Systemic	+/-	[2], [3], [4], [8]
Training, Audit, Feedback	Systemic	+	[2], [3], [4]
Clinic Flow/Staff Motivation	Clinician	+/-	[2], [5], [11]
Expertise in STI	Clinician	+/-	[6], [15]
Electronic Protocols/Guidelines	Clinician	+	[5], [7], [11]
Accessibility/Insurance	Patient	+/-	[9], [12]
Cultural Competence	Patient	-	[5], [16]
Consultation Time/Capacity	Clinician	+/-	[5], [11]
Age/Sexuality	Patient	+/-	[9], [15]
Provider Interactions	Clinician	+/-	[9], [15]

**Awareness/Adherence to Programs**	Both	+/-	[10], [17]
**Partner Knowledge**	Patient	+	[10]
**Clinician Understanding/Benefits**	Clinician	-	[11]
**Patient Preferences**	Patient	-	[11], [15]
**Gender/Ethnicity/Region**	Patient	+/-	[12], [15], [16]
**Reliability/Cost of Test**	Clinician	+	[13]
**Sexual Orientation/Relationship**	Patient	+/-	[14]
**Clinical Discharge/Behaviour**	Patient	+	[15]
**Responsiveness/Ethnicity/Sex**	Clinician	+/-	[16]

Translation process overview

1. The influence matrix catalogs barriers and facilitators to STI testing as identified through a systematic literature review.
2. **Variable quantification**
   a. Variables were defined throughout this process by critically examining what could be parameterised. The final models will have variables which fit their paradigms.
3. **Model integration**
   a. Identified variables were systematically integrated into the SHSM and BBN structures. Placement and interaction of the variables were aligned with the causal diagrams for each model.

References

1. Martin K, Olaru ID, Buwu N, Bandason T, Marks M, Dauya E, Muzangwa J, Mabey D, Dziva Chikwari C, Francis SC *et al*: **Uptake of and factors associated with testing for sexually transmitted infections in community-based settings among youth in Zimbabwe: a mixed-methods study.** *Lancet Child Adolesc Health.* 2021, **5**(2):122-132,10.1016/S2352-4642(20)30335-7.
2. Martin K, Wenlock R, Roper T, Butler C, Vera JH: **Facilitators and barriers to point-of-care testing for sexually transmitted infections in low- and middle-income countries: a scoping review.** *BMC Infect Dis.* 2022, **22**(1):561,10.1186/s12879-022-07534-9.
3. Chesang K, Hornston S, Muhenje O, Saliku T, Mirjahangir J, Viitanen A, Musyoki H, Awuor C, Githuka G, Bock N: **Healthcare provider perspectives on managing sexually transmitted infections in HIV care settings in Kenya: A qualitative thematic analysis.** *PLoS Med.* 2017, **14**(12):e1002480,10.1371/journal.pmed.1002480.
4. Wilkinson A, Ebata A, MacGregor H: **Interventions to Reduce Antibiotic Prescribing in LMICs: A Scoping Review of Evidence from Human and Animal Health Systems.** *Antibiotics (Basel).* 2018, **8**(1):2-2,10.3390/antibiotics8010002.
5. Rana J, Guiang CB, Lisk R, Shahin R, Brunetta J, Mitterni L, Grewal R, Tan DHS, Gilbert M, Yeung A *et al*: **Survey of Health Care Providers' Practices and Opinions Regarding Bacterial Sexually Transmitted Infection Testing Among Gay, Bisexual, and Other Men Who Have Sex With Men.** *Sex Transm Dis.* 2021, **48**(2):94-102,10.1097/OLQ.0000000000001287.
6. Rietmeijer CA: **Improving care for sexually transmitted infections.** *J Int AIDS Soc.* 2019, **22 Suppl 6**(Suppl Suppl 6):e25349,10.1002/jia2.25349.
7. Taylor MM, McClain T, Javanbakht M, Brown B, Aynalem G, Smith LV, Kerndt PR, Peterman TA: **Sexually transmitted disease testing protocols, sexually transmitted disease testing, and discussion of sexual behaviors in HIV clinics in Los Angeles County.** *Sex Transm Dis.* 2005, **32**(6):341-345,10.1097/01.olq.0000154500.01801.db.
8. Ong JJ, Peng M, Zhu S, Lo YJ, Fairley CK, Kidd MR, Roland M, Jiang S, Wong WCW: **Opportunities and barriers to STI testing in community health centres in China: a nationwide survey.** *Sex Transm Infect.* 2017, **93**(8):566-571,10.1136/sextrans-2017-053196.
9. Meanley S, Gale A, Harmell C, Jadwin-Cakmak L, Pingel E, Bauermeister JA: **The role of provider interactions on comprehensive sexual healthcare among young men who have sex with men.** *AIDS Educ Prev.* 2015, **27**(1):15-26,10.1521/aeap.2015.27.1.15.
10. Currie S, Mercer CH, Dunbar KJ, Saunders J, Woodhall SC: **Do healthcare professionals and young adults know about the National Chlamydia Screening Programme? Findings from two cross-sectional surveys.** *International Journal of STD & AIDS.* 2019, **30**(1):72-78,10.1177/0956462418797849.
11. Gaborit L, Polkinghorne B, Marmor A: **How do general practitioners test and treat gonococcal infections in the Australian Capital Territory? Implications

for disease surveillance and control. *Commun Dis Intell (2018).* 2022, **46**10.33321/cdi.2022.46.45.

12. Goyal MK, Witt R, Hayes KL, Zaoutis TE, Gerber JS: **Clinician Adherence to Recommendations for Screening of Adolescents for Sexual Activity and Sexually Transmitted Infection/Human Immunodeficiency Virus**. *The Journal of Pediatrics.* 2014, **165**(2):343-347,10.1016/j.jpeds.2014.04.009.
13. Hsieh YH, Gaydos CA, Hogan MT, Uy OM, Jackman J, Jett-Goheen M, Albertie A, Dangerfield DT, 2nd, Neustadt CR, Wiener ZS *et al*: **What qualities are most important to making a point of care test desirable for clinicians and others offering sexually transmitted infection testing?** *PLoS One.* 2011, **6**(4):e19263,10.1371/journal.pone.0019263.
14. Heller R, Fernando I, MacDougall M: **Factors associated with a clinician's offer of screening HIV-positive patients for sexually transmitted infections, including syphilis**. *Int J STD AIDS.* 2011, **22**(6):351-352,10.1258/ijsa.2010.010410.
15. Anker B, Jaffar S, Patani H, Bristow CC, Sukhija-Cohen AC: **Clinical Factors Associated With Accurate Presumptive Treatment of Neisseria gonorrhoeae Infections in Men Who Have Sex with Men and Transgender Women**. *Clin Infect Dis.* 2021, **73**(9):e3156-e3162,10.1093/cid/ciaa1828.
16. Su JY, Belton S, Ryder N: **Why are men less tested for sexually transmitted infections in remote Australian Indigenous communities? A mixed-methods study**. *Cult Health Sex.* 2016, **18**(10):1150-1164,10.1080/13691058.2016.1175028.
17. Yeung A, Temple-Smith M, Fairley C, Hocking J: **Narrative review of the barriers and facilitators to chlamydia testing in general practice**. *Aust J Prim Health.* 2015, **21**(2):139-147,10.1071/PY13158.
