# Supplementary material for: Policy, practice, and prediction: model-based approaches to evaluating N. gonorrhoeae antibiotic susceptibility test uptake in Australia
Source: BMC Infect Dis. 2024 May 17;24:498. doi: 10.1186/s12879-024-09393-y (PMC11100046; doi:10.1186/s12879-024-09393-y)
Supplement: Supplementary file 2 — Supplementary Material 2 [file 12879_2024_9393_MOESM2_ESM.pdf]

Parameterisation of Bayesian Belief network

The model has been built to model the diagnostic choices required for a clinician to initiate an antibiotic susceptibility test. The diagnostic pathway consists of the following considerations:

1. Clinical experience with the disease [Clinician]
2. Presence of clinical support tools [Clinician]
3. Epidemiological factors of the patient which may present a risk for reinfection/persistence of symptoms with the following parents: [Patient]
    a. Sexual history:
        i. Number of partners
        ii. Sexual orientation of patient
        iii. Past diagnoses of the disease
4. Medication adherence [Patient]
    a. Medication adherence affects the state of persistence of symptoms.

These factors have been translated to the following directed acyclic graph (DAG) based on the influence matrix displayed in supplementary. Relevant variables were selected to create a causal relationship diagram for AST initiation. Expert opinion has been sought for further clarification and refinements to the causal relationship. This can be seen in supplementary fig 1.

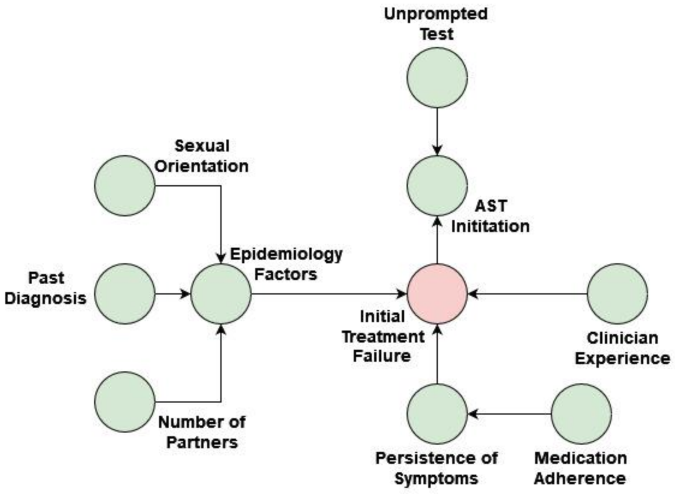

Sup Fig 1 Directed acyclic graph (DAG) presented for antibiotic susceptibility test (AST) initiation for N. gonorrhoeae based on a literature search and expert opinion.

Modelling Notes **Supplementary File 3**

From the DAG, the following model has been created as seen in supplementary fig 2. Parameterisation was completed through expert elicitation and a search for quantitative literature regarding AMR testing and factors implicated in initiation.

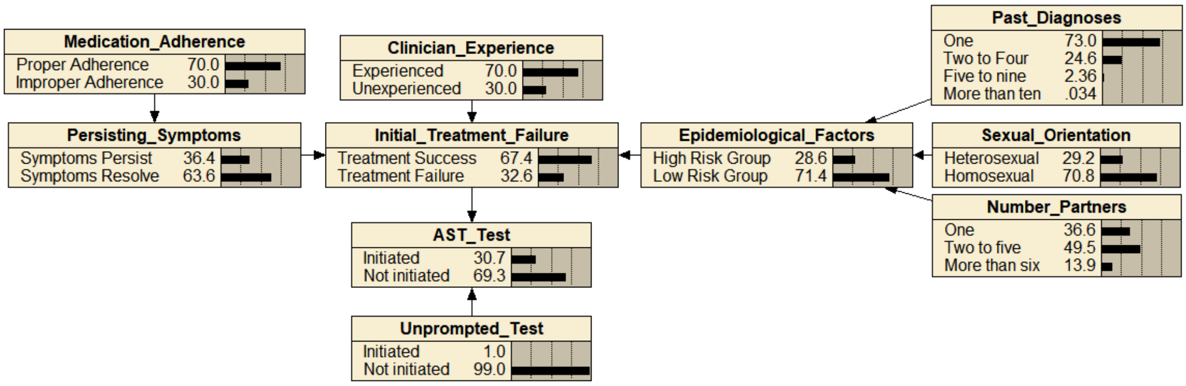

Sup Fig 2 Bayesian belief network diagram for determining antibiotic susceptibility testing (AST)

Description of the model

The logical flow of the model captures the scenario of a clinician determining whether to administer an antibiotic susceptibility test (AST) to a patient with suspected Neisseria gonorrhoeae. The key determinant in administering an AST test to a patient (and subsequently generate an associated resistance surveillance data point) is the initial treatment failing to relieve symptoms. For initial treatment to fail there are both clinician factors and patient level determinants. The clinician factors pertain to clinician experience with the disease. This is inclusive work experience, as a measure of time, and education surrounding the disease. The patient factors pertain to adherence to medication which may cause a persistence in symptoms. Epidemiological factors are also a consideration. Higher risk of disease presence and transmission may cause initial treatment to fail. Past diagnoses, sexual orientation, and number of partners within a 30-day period are considerations made within the model.

Patient factors – Epidemiological Factors

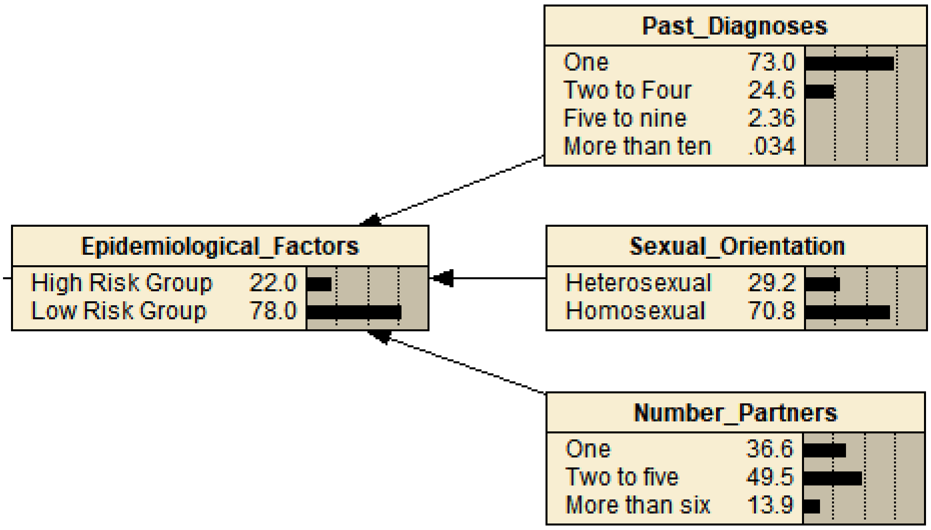

Sup Fig 3 Epidemiological factors within the Bayesian belief network (BBN)

Epidemiological factors of an individual play an important part of diagnoses and determination of a patient's risk for reinfection/resistance [1, 2].

- **Past Diagnoses**

Past diagnoses has been linked to an increased likelihood of an individual having a resistant isolate [3]. Categories used within Allen, et al. [3] have been used to parameterise the model. The prevalence/proportion of each group have been used to populate the past diagnoses table. Past diagnoses may indicate a great susceptibility to reinfection or persistence of N. gonorrhoea symptoms that has not been properly managed.

- **Number of partners**

Number of partners has also been identified to be a risk factor for resistance through increased risk of N. gonorrhoea [3, 4]. Prevalence of the groups have been taken from Allen, et al. [3]. Unknown parameters were not counted as due to the complexity that would be added to the model. Number of partners relates to the likelihood that an individual will contract N. gonorrhoea. General literature surrounding sextually transmitted diseases suggests an increase number of partners increases the risk of disease prevalence [5].

- **Sexual Orientation**

Modelling Notes **Supplementary File 3**

Sexual orientation has been identified as a potential risk factor that increases the likelihood of antimicrobial resistance being present [4-6]. Prevalence of N. gonorrhoea within Australia has been used to parameterise the variable.

The following probability table was generated from expert elicitation using best-worst scenario as detailed within Cain [7].

Supplementary Table 1 Conditional probability table (CPT) for epidemiological factors in the initiation of AST for N. gonorrhoeae

Sexual Orientation	Past Diagnoses	Number Partners	High Risk Group	Low Risk Group
Heterosexual	One	One	5	95
Heterosexual	One	Two to five	20	80
Heterosexual	One	More than six	35	65
Heterosexual	Two to Four	One	30	70
Heterosexual	Two to Four	Two to five	45	55
Heterosexual	Two to Four	More than six	60	40
Heterosexual	Five to nine	One	65	35
Heterosexual	Five to nine	Two to five	80	20
Heterosexual	Five to nine	More than six	95	5
Heterosexual	More than ten	One	75	25
Heterosexual	More than ten	Two to five	90	10
Heterosexual	More than ten	More than six	95	5
Homosexual	One	One	10	90
Homosexual	One	Two to five	25	75
Homosexual	One	More than six	40	60
Homosexual	Two to Four	One	40	60
Homosexual	Two to Four	Two to five	55	45
Homosexual	Two to Four	More than six	70	30
Homosexual	Five to nine	One	75	25
Homosexual	Five to nine	Two to five	85	15
Homosexual	Five to nine	More than six	95	5
Homosexual	More than ten	One	80	20

**Homosexual**	More than ten	Two to five	90	10
**Homosexual**	More than ten	More than six	95	5

Adherence to treatment

Treatment adherence is an important consideration made within the model. Failure to adhere to treatment regimens (i.e., misuse of antibiotics) has been implicated in the development of resistance [8, 9]. Moreover, the misuse of antibiotics and subsequently development of resistance, may cause a persistence the symptoms for N. gonorrhoeae [10]. Persistence of symptoms is an indicator is a strong indicator that initial treatment has failed or not worked as intended which prompts further investigation.

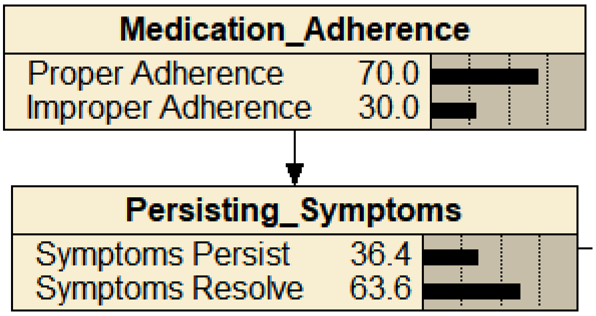

Sup Fig 4 Medication adherence and persistence of symptoms as variable of the BBN

Proper adherence has been estimated from general figures regarding any medication. The variable encompasses any medication as derived estimates [11]. There is sparse literature regarding medication adherence directly related to N. gonorrhoeae. However, estimates can be justified by the comparability of conditions with oral use of antibiotics.

The conditional probability table for medication adherence is as shown in supplementary table 2. The table was populated using Eells, et al. [12] odds ratios regarding medication adherence.

Supplementary Table 2 Medication adherence and persistence of symptoms conditional probability table (CPT)

Medication Adherence	Symptoms Persist	Symptoms Resolve
Proper adherence	18	84
Improper Adherence	84	16

Clinician Experience

Clinician experience is not presupposed on the notion of AMR awareness. Although it is a possibility, the model does not consider this as a possibility. The model views clinician experience as a factor that lowers the probability of initial treatment failure. Indeed, an increase in clinician experience will lower the probability of initial treatment failure and thus lower the probability of an individual having an AST initiated.

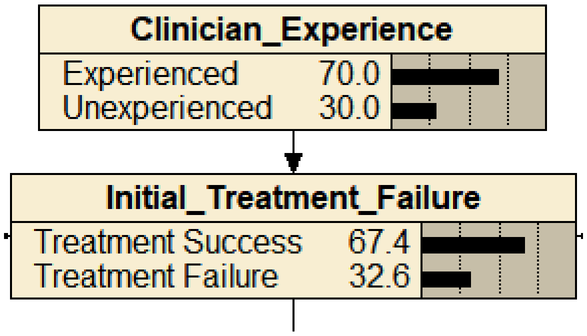

Sup Fig 5 Clinician experience within the BBN.

Clinician experience and its parameterisation is highly contentious. Currently, there is no literature which quantifies how much of the clinician population is "experienced" with regards to sexual health or N. gonorrhoeae. Therefore, an estimate has been made regarding the steady state proportion to conservatively estimate 70% of the population is experienced. This could be through general population subspeciality (i.e., General practice, infectious disease medicine, sexual health medicine) and/or further education/training in the area.

Unprompted test

Upon expert elicitation, there was the revelation of systematic testing factors which could deterministically bypass all other clinical factors i.e., randomised testing for recently visited to Southeast Asia or systematic testing. Though it is rare, it could be a possibility. If an unprompted test is initiated, then there should be very little chance that there is a no test initiated.

Modelling Notes **Supplementary File 3**

Initial treatment failure

Initial treatment failure is the main node which largely influences whether nor not an AST is initiated. The following CPT has been made using expert elicitation in supplementary table 3.

Supplementary Table 3 Conditional probability table of initial treatment failure with parent nodes and probabilities out the outcome

Persisting Symptoms	Clinician Experience	Epidemiological Factors	Treatment Success	Treatment Failure
Symptoms Persist	Experienced	High Risk Group	15	85
**Symptoms Persist**	Experienced	Low Risk Group	30	70
**Symptoms Persist**	Unexperienced	High Risk Group	5	95
**Symptoms Persist**	Unexperienced	Low Risk Group	15	85
**Symptoms Resolve**	Experienced	High Risk Group	90	10
**Symptoms Resolve**	Experienced	Low Risk Group	95	5
**Symptoms Resolve**	Unexperienced	High Risk Group	90	10
**Symptoms Resolve**	Unexperienced	Low Risk Group	95	5

Initiation of AST

Initiation of AST is the target node. The conditional probability table is made as such in supplementary table 4. This has been parameterised by clinician/expert input.

Supplementary Table 4 Conditional probability table of initiating antibiotic susceptibility testing based on initial treatment failure.

Initial Treatment Failure	Unprompted Test	Initiated	Not initiated
Treatment Success	Initiated	99	1
**Treatment Success**	Not initiated	1	99
**Treatment Failure**	Initiated	99	1

Treatment Failure	Not initiated	90	10

Model Assumptions

There are key model assumptions that must be acknowledged in the interpretation of model results. These are displayed in supplementary table 5 for the core assumptions of the model and the variables.

Supplementary Table 5 Key model and variable assumptions of the model

Key model assumptions	
**Model assumptions**	• The model assumes the following weighting for order the variables. The ranking is as follows:<br>o 1) Past diagnoses<br>o 2) Sexual Orientation<br>o 3) Number of Partners<br>• Past diagnoses have the greatest weighting due to the possibility of reinfection/persistence of symptoms as an indication of resistance/failure of past treatment.<br>• Number of partners is the lowest weighted parameter as the variable includes multitudes of complexity (i.e., safe sex measures, gender/sexual orientation of partners, type of sexual act)<br>• Sexual orientation has been given two classifications for simplicity.<br>o Homosexual: Encompasses men who have sex with men (MSM) and bisexual men.<br>o Heterosexual: Encompasses men who have sex with women (MSW)<br>• Classification as high epidemiological risk would suggest there to be a significant

	possibility that the individual is carrying a resistant organism to treatment (dictated by number of infections, sexual orientation, and number of partners)<br>• The model also assumes that the location of the infection does not matter.<br>• Accessibility to resources for AST are also not considered within the model. Therefore, all access to resources is deemed to be uniform across all settings. This is due to limitations in the literature.<br>• All patients are compliant. All reported medication adherence is reflective of actual behaviour.<br>• Diagnoses are assumed to be accurate (100% accuracy) with the only factor left to consider is resistance.<br>• Uniformity of clinical practice. There exists variation. However, for parsimony, uniformity of clinical practice has been assumed. There are nuances, with clinician experience depending on location.<br>• Stability in epidemiological factors is assumed and is not subject to rapid changes.<br>• The model does not account for healthcare system factors which may affect clinical experience probabilities. This is assumed to be the same across locations.<br>• Behaviours of the patients are assumed to be sufficient to gain enough information regarding the reporting sexual history and sexual behaviours of the individual. This assumption refers to patient homogeneity and has been

	made for generalisability.
**Variable assumptions**	
**Past diagnoses**	• Past diagnoses would assume an increased chance of having a resistant organism. The clinician is assumed to have perceived it this way with recurrent infections (or may be perceived as the same presentation twice which would be a persisting infection).
**Sexual Orientation**	• Epidemiological literature has suggested that N. gonorrhoea has exacerbated burden amongst MSM populations [13].<br>• Moreover, MSM have exacerbated risk for resistance [13].
**Number of partners**	• Number of partners is deemed to be lowest due to multitude of factors involved (i.e., safe sex and acts) that cannot be parameterised accurately by the model.
**Unprompted test**	• Unprompted tests could refer to systematic tests (i.e., every 5th isolate should be tested) or the clinician has decided to test regardless of the presentation

Sensitivity Analyses

Supplementary Table 6 Sensitivity analysis results with the target node AST Test

Node	Mutual Info	Percent	Variance of Beliefs
**AST Test**	0.86174	100	0.2036357
**Initial Treatment Failure**	0.60778	70.5	0.1554389
**Persisting Symptoms**	0.30910	35.9	0.0847199
**Medication Adherence**	0.12019	13.9	0.0355356
**Epidemiological Factors**	0.00329	0.382	0.0009463

Clinician Experience	0.00127	0.147	0.0003624
Past Diagnoses	0.00035	0.0404	0.0000992
Unprompted Test	0.00018	0.0205	0.0000522
Number Partners	0.00017	0.0193	0.0000471
Sexual Orientation	0.00001	0.00152	0.0000037

Supplementary Table 7 Sensitivity Analysis on the node Initial Treatment Failure

Node	Mutual Info	Percent	Variance of Beliefs
Initial Treatment Failure	0.91092	100	0.2197696
AST Test	0.57890	63.6	0.1621328
Persisting Symptoms	0.41768	45.9	0.1197826
Medication Adherence	0.16088	17.7	0.0502426
Epidemiological Factors	0.00432	0.475	0.0013379
Clinician Experience	0.00167	0.183	0.0005124
Past Diagnoses	0.00046	0.0501	0.0001403
Number Partners	0.00022	0.024	0.0000666
Sexual Orientation	0.00002	0.00189	0.0000052
Unprompted Test	0.00000	0	0.0000000

Supplementary Table 8 Sensitivity analyses on the node Epidemiological Factors

Node	Mutual Info	Percent	Variance of Beliefs
Epidemiological Factors	0.86354	100	0.2042162
Past Diagnoses	0.07099	8.22	0.0214132
Number Partners	0.03629	4.2	0.0101687
Initial Treatment Failure	0.00432	0.501	0.0012432
AST Test	0.00329	0.381	0.000949

Sexual Orientation	0.00288	0.333	0.0007984
Unprompted Test	0	0	0
Clinician Experience	0	0	0
Persisting Symptoms	0	0	0
Medication Adherence	0	0	0

References

1. Sarenje KL, Ngalamika O, Maimbolwa MC, Siame A, Munsaka SM, Kwenda G: **Antimicrobial resistance of Neisseria gonorrhoeae isolated from patients attending sexually transmitted infection clinics in Urban Hospitals, Lusaka, Zambia.** BMC Infect Dis 2022, **22**(1):688. 10.1186/s12879-022-07674-y
2. Whelan J, Abbing-Karahagopian V, Serino L, Unemo M: **Gonorrhoea: a systematic review of prevalence reporting globally.** BMC Infect Dis 2021, **21**(1):1152. 10.1186/s12879-021-06381-4
3. Allen H, Merrick R, Ivanov Z, Pitt R, Mohammed H, Sinka K, Hughes G, Fifer H, Cole MJ: **Is there an association between previous infection with Neisseria gonorrhoeae and gonococcal AMR? A cross-sectional analysis of national and sentinel surveillance data in England, 2015-2019.** Sex Transm Infect 2023, **99**(1):1-6. 10.1136/sextrans-2021-055298
4. Mortimer TD, Pathela P, Crawley A, Rakeman JL, Lin Y, Harris SR, Blank S, Schillinger JA, Grad YH: **The Distribution and Spread of Susceptible and Resistant Neisseria gonorrhoeae Across Demographic Groups in a Major Metropolitan Center.** Clin Infect Dis 2021, **73**(9):e3146-e3155. 10.1093/cid/ciaa1229
5. Callander D, Guy R, Fairley CK, McManus H, Prestage G, Chow EPF, Chen M, Connor CCO, Grulich AE, Bourne C, Hellard M, Stoove M, Donovan B, Collaboration A: **Gonorrhoea gone wild: rising incidence of gonorrhoea and associated risk factors among gay and bisexual men attending Australian sexual health clinics.** Sex Health 2019, **16**(5):457-463. 10.1071/SH18097
6. Jacobsson S, Cole MJ, Spiteri G, Day M, Unemo M, Euro GN: **Associations between antimicrobial susceptibility/resistance of Neisseria gonorrhoeae isolates in European Union/European Economic Area and patients' gender, sexual orientation and anatomical site of infection, 2009-2016.** BMC Infect Dis 2021, **21**(1):273. 10.1186/s12879-021-05931-0
7. Cain J: **Planning Improvements in Natural Resources Management**, vol. 124; 2001.
8. Baumgartner PC, Haynes RB, Hersberger KE, Arnet I: **A Systematic Review of Medication Adherence Thresholds Dependent of Clinical Outcomes.** Front Pharmacol 2018, **9**:1290. 10.3389/fphar.2018.01290
9. Costelloe C, Metcalfe C, Lovering A, Mant D, Hay AD: **Effect of antibiotic prescribing in primary care on antimicrobial resistance in individual patients: systematic review and meta-analysis.** BMJ 2010, **340**:c2096. 10.1136/bmj.c2096
10. La Rosa R, Johansen HK, Molin S: **Persistent Bacterial Infections, Antibiotic Treatment Failure, and Microbial Adaptive Evolution.** Antibiotics (Basel) 2022, **11**(3):419. 10.3390/antibiotics11030419
11. Glombiewski JA, Nestoriuc Y, Rief W, Glaesmer H, Braehler E: **Medication adherence in the general population.** PLoS One 2012, **7**(12):e50537. 10.1371/journal.pone.0050537
12. Eells SJ, Nguyen M, Jung J, Macias-Gil R, May L, Miller LG: **Relationship between Adherence to Oral Antibiotics and Postdischarge Clinical Outcomes among Patients Hospitalized with Staphylococcus aureus Skin Infections.** Antimicrob Agents Chemother 2016, **60**(5):2941-2948. 10.1128/aac.02626-15
13. Abraha M, Egli-Gany D, Low N: **Epidemiological, behavioural, and clinical factors associated with antimicrobial-resistant gonorrhoea: a review.** F1000Res 2018, **7**:400. 10.12688/f1000research.13600.1
