# Supplementary material for: Policy, practice, and prediction: model-based approaches to evaluating N. gonorrhoeae antibiotic susceptibility test uptake in Australia
Source: BMC Infect Dis. 2024 May 17;24:498. doi: 10.1186/s12879-024-09393-y (PMC11100046; doi:10.1186/s12879-024-09393-y)
Supplement: Supplementary file 3 — Supplementary Material 3 [file 12879_2024_9393_MOESM3_ESM.pdf]

Model Description

The SHSM is a probabilistic model that represents the interactions between a synthetic population with N. *gonorrhoeae* (with a resistant isolate) and the healthcare system (clinicians). Agents within the SHSM model are the clinicians. Clinicians will have different characteristics and behaviours on the basis. It is designed to understand and predict the initiation of AST, which is critical to generating a data point for N. *gonorrhoeae* AMR surveillance. This model follows the following visual schematic. Methodological clarification can be seen within the methods section of the paper.

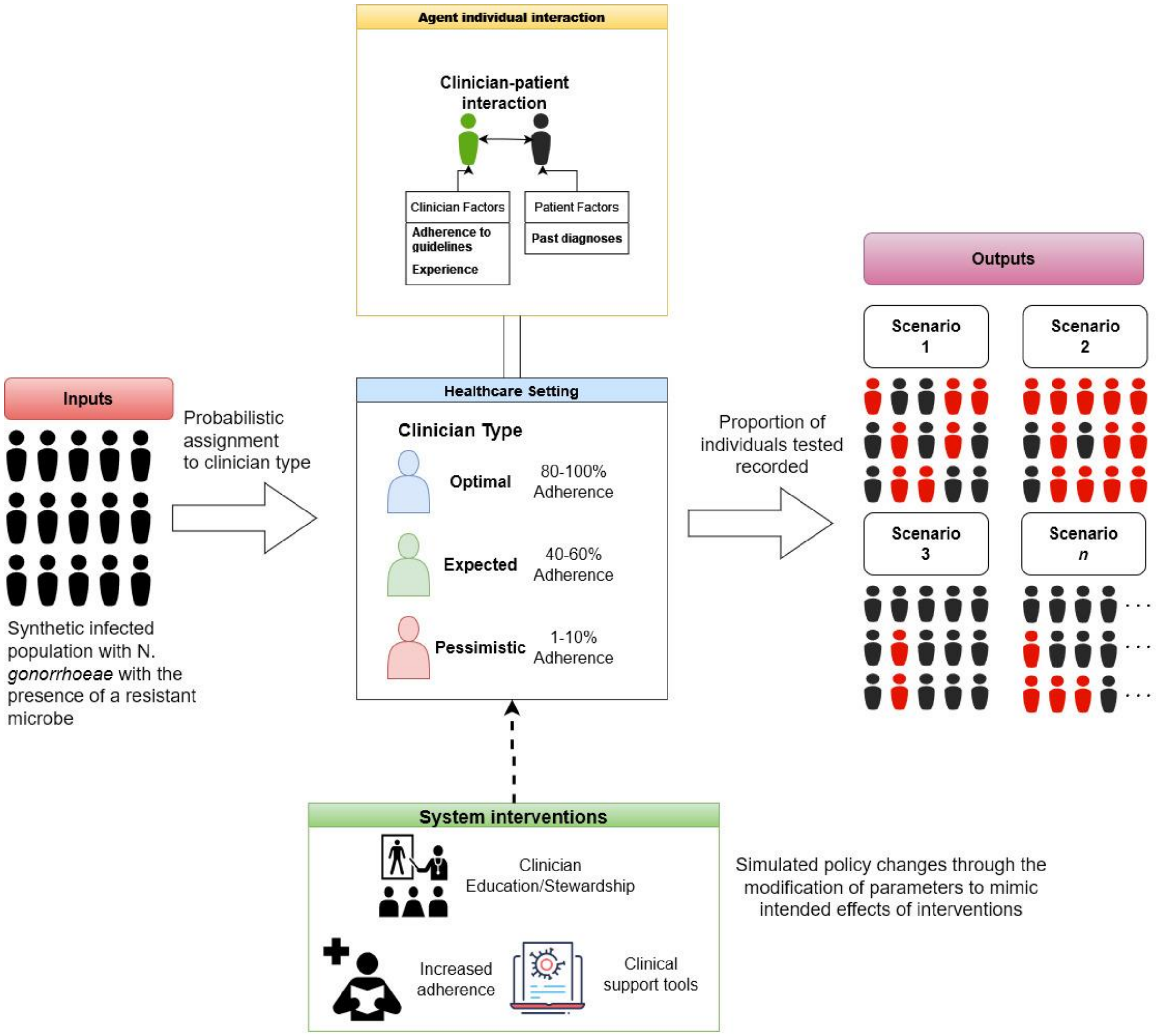

Figure 1 Visual schematic of the model with the synthetic population and interaction between clinicians.

The choice for this is predicated on the following causal pathway. Whilst all factors are important, there they can be encompassed by

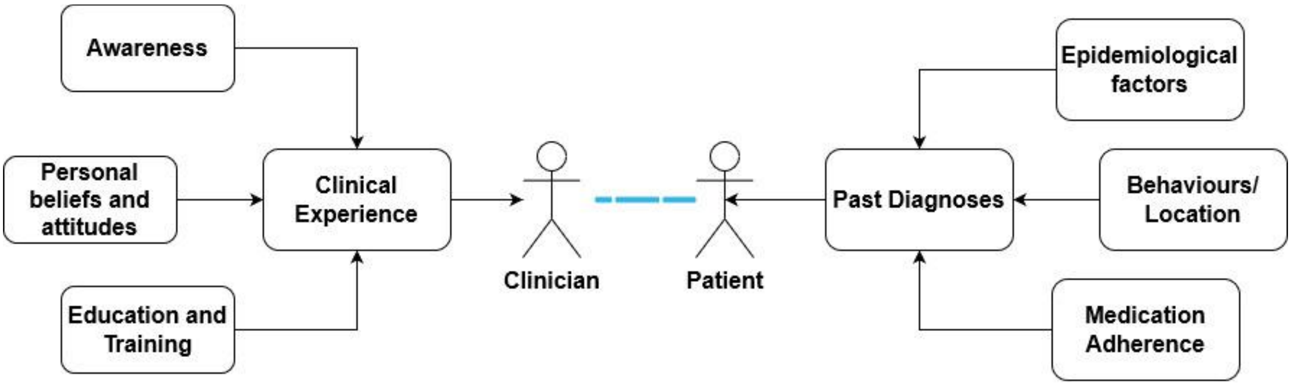

Figure 2 Causal pathway diagram with presenting factors encompassed by the model.

The following decisions were made based on this model's fundamental paradigm. Whilst there are deterministic factors, the event of an individual being tested is the product of two independent events. Certainly, it is possible for an individual to be tested regardless of they pose no risk. The latter is also true. It is possible for an individual to pose high risk of resistance, yet have no test initiated to determine resistance status.

In the model, past diagnoses are deemed to be a sufficient indicator for an individual's risk of having a resistant isolate. It is hypothesised, based on past literature, to be the product of all other important factors. That includes adherence to therapeutic regimes, behaviours of the individual, and other epidemiological factors (socio-demographic information). These contribute to the outcome for which the clinician will perceive past diagnoses. If there is a past diagnosis it is treated in a similar manner to a recurrent/persistent infection. Clinician experience is difficult to parameterise. However, for the achievement of parsimony, Clinical experience is deemed to be in align with their training, personal beliefs, and awareness of AMR. This is hypothesised in the model to make a clinician more likely to test. The interaction is modelled by two independent events.

$$\Pr \text{Test}|\text{Risk} = \Pr \text{Test} \cdot \Pr \text{Risk}$$

Table of variables and description

Supplementary Table 1 Variables, assumptions and overview of the variables included within the model

Variable	Assumptions	Overview
**Sex (Male, Female)**	The population is dynamically divided into male and female categories, reflecting natural population proportions.	Used to segregate population data by gender, which affects the distribution of other health and behavioural variables.
**MSM Status**	A subset of the male population is designated as Men who have Sex with Men (MSM), which is associated with higher risk factors for certain health issues.	Important for identifying high-risk groups within the male population, particularly for STIs and HIV.
**Indigenous Status**	Proportionally represents the Indigenous population within the dataset, acknowledging variations in health outcomes and access to services.	Helps to address disparities in health status and outcomes, and tailor health interventions appropriately.
**Diagnoses**	Categorized past diagnoses to stratify the population by health risk related to infection recurrence or resistance.	Critical for assessing individual health risks and predicting the need for advanced medical interventions such as AST.
**HIV Status**	Assigned specifically within the MSM population to account for a higher prevalence of HIV, influencing health management strategies.	Provides insights into comorbidities that may affect treatment plans and health surveillance.
**Age Classification**	The population is divided into age groups to reflect differing risk behaviours and susceptibility to health conditions.	Used to model and understand age-related differences in health status, behaviour, and healthcare needs.
**Sexual Partners**	Number of sexual partners is used as an indicator of sexual behaviour risk, with different thresholds set for males and females.	Impacts assessments of STI risks and guides interventions in sexual health education and prevention.
**Clinician Experience**	Experience levels among clinicians are modelled to affect the probability of initiating diagnostic tests, with a range	Influences the quality of patient care and decision-making in clinical settings, particularly in

	suggesting variability in expertise and confidence.	diagnosing and managing infections.
**Clinical Decision Support Tools**	The effectiveness of these tools is assumed to enhance clinician performance, especially in complex diagnostic scenarios.	Aids in improving diagnosis accuracy, treatment decisions, and overall patient outcomes through enhanced data integration and real-time decision support.
**Patient Risk (θ)**	Derived from a combination of individual health data and external factors, converted into a risk score that influences testing likelihood.	Quantifies the cumulative risk of a patient needing specific health interventions, crucial for targeted testing and treatment strategies.
**Clinician Test Score (τ)**	Combines clinician experience and the influence of decision support tools into a single metric that determines their likelihood to initiate testing.	Reflects the interplay of clinician capability and technological aid in the decision-making process for initiating tests.
**Testing Probability (λ)**	The final probability that a test will be conducted, based on the interaction of patient risk and clinician readiness, modelled probabilistically.	Determines the likelihood of a patient being tested, which is critical for accurate disease surveillance and management.
**Testing Decision**	Based on the testing probability, this binary outcome decides whether a patient is tested or not, simulating the real-world decision-making process in healthcare settings.	Directly affects the dataset's representation of testing rates, providing a basis for evaluating healthcare efficacy and resource allocation.
**Adherence (α)**	Adherence is dynamically set and probabilistic rather than deterministic. Adherence assumes that this is a probabilistic modifier for clinician behaviour for example adherence of $\alpha = 1.00$ would indicate that based on a clinician's own presumptions and practice, they would always adhere to that behaviour. Values below indicate there would be more randomness.	Adherence (α) quantifies the likelihood that clinicians will follow specific guidelines or treatment protocols. It is a critical variable that influences the model's simulation of clinical decisions and behaviours, thus affecting the outcomes of interest such as the initiation of antibiotic susceptibility testing (AST). By modelling adherence probabilistically, the model can more accurately reflect the nuances and variability of clinical practice, capturing how different levels of adherence

		impact the health care process and patient outcomes.

At baseline, the split for clinician adherence is 70%/25%/5% (Moderate, High, and Low Adherence) defined later under **Clinician Experience** with their values. This scenario, at baseline, is a conservative estimate. A high adherence scenario is the assumption that most clinicians are willing to follow the set guidelines for testing and may not rely on intuitive practice. This is set at 5%/90%/5% whereby 5% are moderately adhering, 90% are highly adhering and 5% have low adherence.

Population parameterisation

Population parameterisation is reflected by results indicated by past literature [1]. The distribution of males and females (with MSM subset) is presented as the potential population parameters to must be considered. Population proportions are obtained from the reports outlined by King, et al. [2]. Males who have sex with males (MSM) is given by estimates in literature by Mauck, et al. [3]. The number of sexual partners for the population parameters have been derived from Allen, et al. [1] which encompasses self-reported number of partners $\leq$ 3 months.

Supplementary Table 2 Population parameters for the generated tested population

	Male (MSM) (3.6% - 7.0%) [3]	Male (n = 49.8%)	Female (n = 50.2%)
**Sexual Partners**			
0-1	12.1	28.2	38.7
2-5	22.9	37.0	22.5
6+	9.3	4.1	2.1
**Previous diagnosis**			
1	63.3	91.0	80.4
2-4	33.1	9.0	18.7
5-9	3.6	0	0.9
> 10	0.1	0	0.0

Notes:

Modelling Notes **Supplementary File 2**

- Look at Australian distribution of HIV status (% infected as population parameter but also include wider range of estimates)
- The percentages for males and females need to be changed so that they reflect the epidemiology of N. gonorrhoeae (70-30% split).
- Distributions of N. *gonorrhoeae* will be used to inform weights.

Odds ratio weights for patient risk

Patient risks (w_i) are given by the following table derived from literature analysing the associations between epidemiological characteristics and N. *gonorrhoeae* resistance prevalence found in Allen, et al. [1]. Within Allen, et al. [1], the study looks at the association, that with a certain exposure (i.e., number of previous diagnoses within the past 3 years) will have return a resistant N. *gonorrhoeae* specie. For the purposes of the study, **Ceftriaxone**, odds ratios (OR) have been used as they are the first line of treatment within Australia. The OR represents the association between the number of diagnoses with the probability that the N. *gonorrhoeae* isolate returns elevated minimum inhibitory concentration (MIC). Adjusted ORs has been utilised as this already accounts for the increased/decreased risk presented by other confounders.

Supplementary Table 3 Univariate and multivariate odds ratios used for Ceftriaxone, Cefixime, and Azithromycin for MSM population.

Number of diagnoses	Lower 95%	OR	Upper 95%	Lower	Adjusted[a] OR	Upper	Distribution
**Ceftriaxone**							
1	1.00	1.00	1.00	0	1.00	0	$\text{Uniform}(0,1)$
2-4	1.08	1.58	2.32	1.07	1.59	2.37	$\text{PERT}(1.07,1.59,2.37)$
5-9	0.59	1.49	3.78	0.71	1.85	4.82	$\text{PERT}(0.71,1.85,4.82)$
> 10[†]	0.48	1.84	5.14	0.636	2.32	6.55	$\text{PERT}(0.636,2.32,6.55)$

[a] Study indicates that there is adjustment for concurrent STI diagnosis and year of NG diagnosis. However, for practicality, this is the odds ratio used in weighting if there is another concurrent STI.

Odds ratios for non-MSM populations are given in Supplementary Table 3. However, as there is insufficient data for diagnosis categories above 2-4, odds ratios from the MSM table have been used as estimates for 5-9 and 10+.

Supplementary Table 4 Univariate results showing the OR among Neisseria gonorrhoeae isolates from heterosexual men and all women.

Number of diagnoses	Lower 95%	OR	Upper 95%	Distribution
1	1.00	1.00	1.00	Uniform(0,1)
2-4	0.37	0.94	2.37	PERT(0.37,0.94,2.37)
5-9[†]	0.71	1.85	4.82	PERT(0.71,1.85,4.82)
> 10[†]	0.636	2.32	6.55	PERT(0.636,2.32,6.55)
Number of diagnoses within the parameters are limited between 1 to 4 diagnoses as per Allen, et al. [1].<br>[†] Values estimated from MSM groups due to insufficient data.				

Clinician Experience

Clinician experience must be estimated. In this case, a conservative estimated was used for baseline simulations. Depending on the clinician in the mode, there could be an increase of 50% or a decrease in 50% in probability of initiating an AST. Therefore, the model parameter for clinician experience z_i was:

$$z_i \sim \text{PERT } 0.50{,}1.00{,}1.50$$

All Clinicians within the model are expected to have a clinician/clinical experience value based on this. Further improvements can be seen in table 4 with the addition of electronic clinical support tools. The weights with electronic clinical decision support tools are derived from Goyal, et al. [4].

Clinician Adherence

For Clinician A

- Modelled as typical adherence to potential clinical guidelines.
- 70% percent of the population will be assigned clinician A initially.
    - Adherence value would be:

$$\alpha \sim \mathrm{U}(0.4{,}0.6)$$

For Clinician B

- Modelled as high adherence to potential clinical guidelines.
- 25% percent of the population will be assigned to clinician B initially.
    - Adherence value would be:

$$\alpha \sim \mathrm{U}(0.8{,}1.0)$$

For Clinician C

- Modelled as low adherence to potential clinical guidelines.
- 5% percent of the population will be assigned to clinician C initially.
    - Adherence parameter would be:

$$\alpha \sim \mathrm{U}(0.01{,}0.10)$$

Individual clinician factor improvements

Modification population parameters are done to simulate systematic changes within the system. The following tables provide the outlines for model modification. Implementation of a clinical decision-making tool is presented in supplementary table 4 on how it will affect the model. This will affect different proportions of the population to simulate implementation to the population. The cases are:

1. 10-25% of the clinician population will have the implementation tool.
2. 26-50% of the clinician population will have the implementation tool.
3. 51-75% of the clinician population will have the implementation tool.
4. 76-100% of the clinician population will have the implementation tool.

The assumption for uptake is explicitly made. The adherence parameter α is the modifier used to encapsulate any potential indiscretions regarding clinical uptake and usage i.e., low adherence clinicians may seldomly use the tool which would dampen its benefit (but may still provide a benefit). This would lead to the following model equation.

$$\phi_b\ \tau_i = \frac{1}{1 + e^{-\alpha \cdot \tau\ y_i, z_i}} \quad \text{where} \quad \tau\ y_i, z_i = \sum_{i=1}^{2} \text{Experience} + \text{Tool}$$

Supplementary Table 5 Electronic clinical decision-making tools for clinician improvement in the simulated model with its distribution

Factor	Lower 95%	Odds	Upper 95%	Distribution
Electronic Clinical Decision Support Tools [4]	1.1	2.0	3.8	PERT(1.1,2.0,3.8)

Within the R file, the improvement is made through the modification of

```
# Clinician tau value
tau_i = patients_df$clinknow + patients_df$eclintool
```

In simulating the change with tool at different proportions

```
# Calculate the number of rows for support tool "present"
# Modify this value to change proportions * 0.10 - 0.25 presented as an example here
  num_present <- round(runif(1, 0.10* num_rows, 0.25 * num_rows))


  # Function to calculate 'eclintool'
  calculate_eclintool <- function(support_tool) {
    if (support_tool == "present") {
      return(rpert(1, min = 1.1, mean = 2, max = 3.8))
    } else if (support_tool == "absent") {
      return(0)
    } else {
      return(NA)  # Handle other cases if needed
    }
  }
```

Improvements in clinician knowledge is done through the modification of the parameter for clinician knowledge.

```
# Education/experience of clinician (estimation)
# Presented here is improvements of 10-25% or anywhere between 1.1 to 1.25
  patients_df$clinknow <- rpert(nrow(patients_df),min = 0.5 *runif(1, min = 1.10, max = 1.25),
            mean = 1.0 *runif(1, min = 1.10, max = 1.25), max = 1.5 *runif(1, min = 1.10, max = 1.25))
```

The scenarios are as follows:

1. 10 – 25% improvement
2. 26 – 50% improvement
3. 51 – 75% improvement
4. 76 – 100% improvement

Dual scenarios are then presented by modifying both parameters in permutations.

Risk-conversion function for clinician experience

The risk conversion functions have inherent assumptions that must be acknowledged. Given the following functions, to convert odds ratios to a risk, the unadjusted magnitude of odds ratios was used. For clinician experience, as this was an estimated parameter, the distribution was symmetrical.

$$\phi_b \ \tau_i \ = \frac{1}{1 + e^{-\alpha \cdot \tau \ y_i, z_i}}$$

The following distribution displayed is the baseline distribution used.

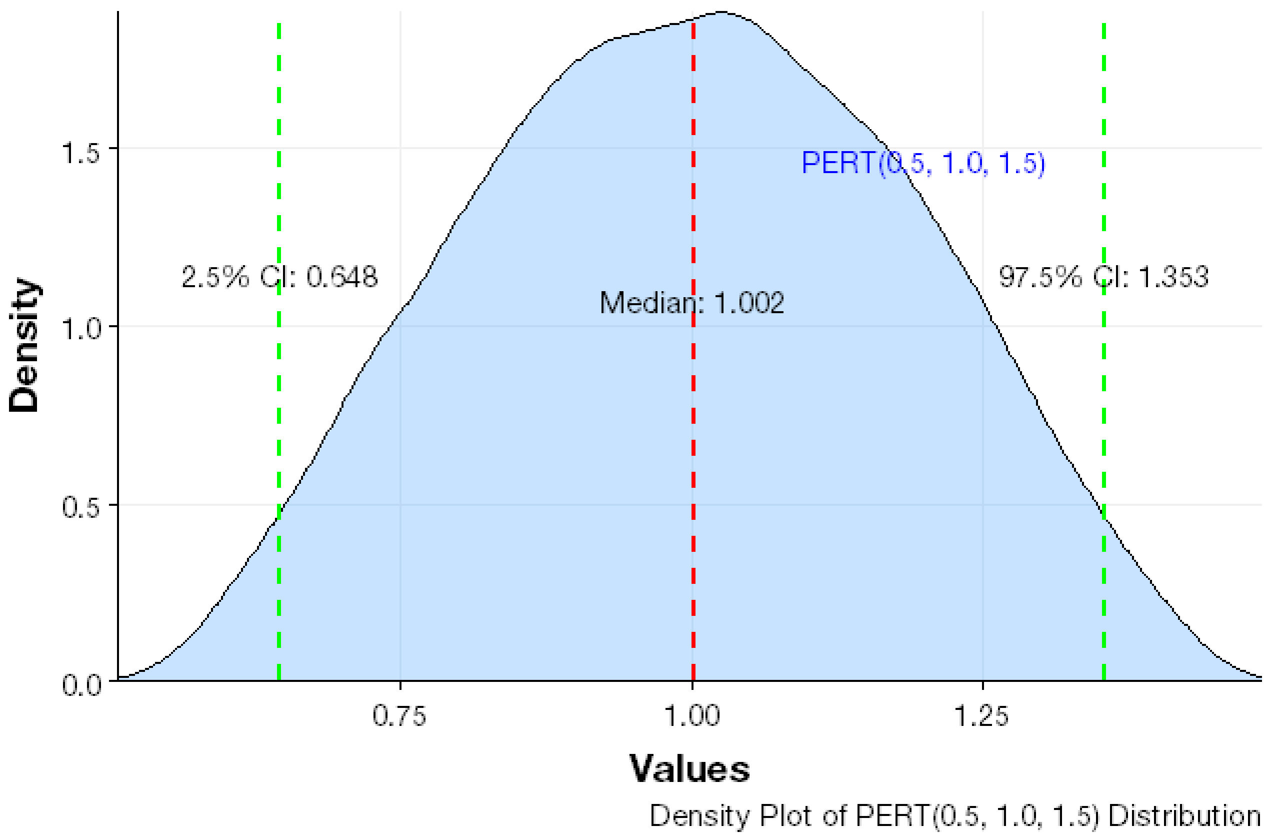

Figure 3 Baseline PERT distribution to model clinician experience

As the distribution chosen is symmetrical, the probability for improvement in clinical care should be approximately the same as detriments to clinical care. The values of the distribution are skewed towards the higher magnitudes of detrimental care as opposed to improvements. For $\tau_i < 1$, we define τ_i as $\tau = -\tau_i$. For instance, we take two instances with a fixed value of $\alpha = 0.70$ with $\tau_i = -0.50$ and $\tau_i = -0.90$ when $\tau_i < 1$

$$\phi_b \ \tau_i \ = \frac{1}{1 + e^{-0.7 \cdot -0.5}} = 0.413 \text{ and } \frac{1}{1 + e^{-0.7 \cdot -0.9}} = 0.347$$

Within the distribution specified, values of $\tau_i = 0.90$ have a greater density than $\tau_i = 0.50$. This skewing of the magnitude is an inherent assumption made within clinician experience to reflect conservatism in estimating clinician behaviour. The following figure demonstrates the effect increasing τ_i has on the overall risk $\phi_b \ \tau_i$. This following

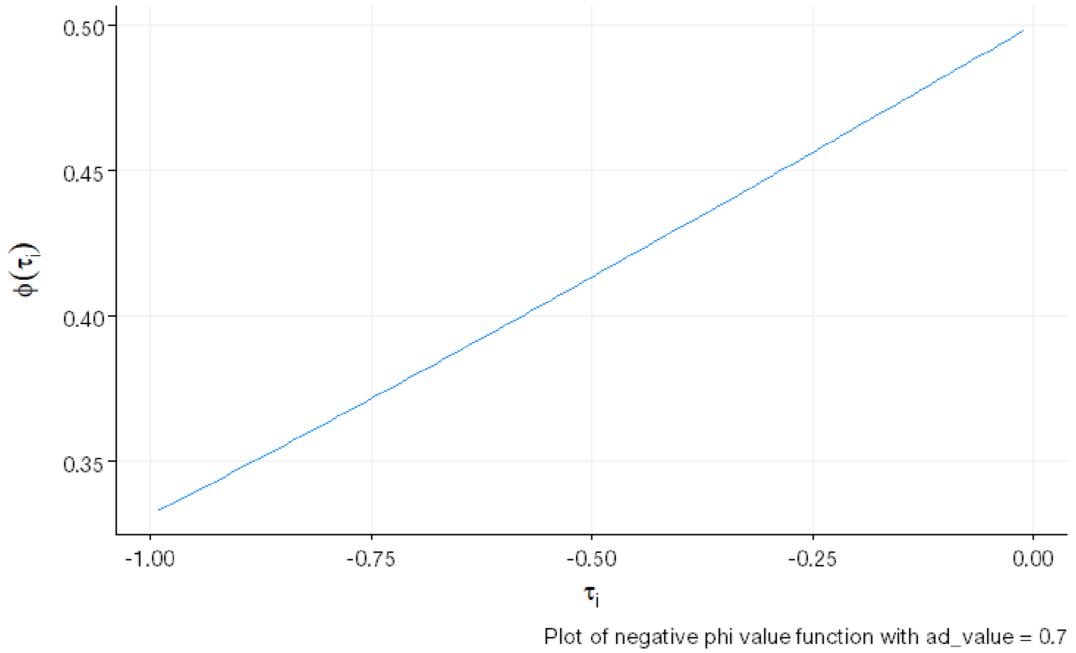

Figure 4 Differing values of τ_i and their respective ϕ value.

The decision to incorporate and interpret odds ratios as reductions (i.e. 0.92 is an 8% reduction is) is shown in direct inversion in as presented in figure 5.

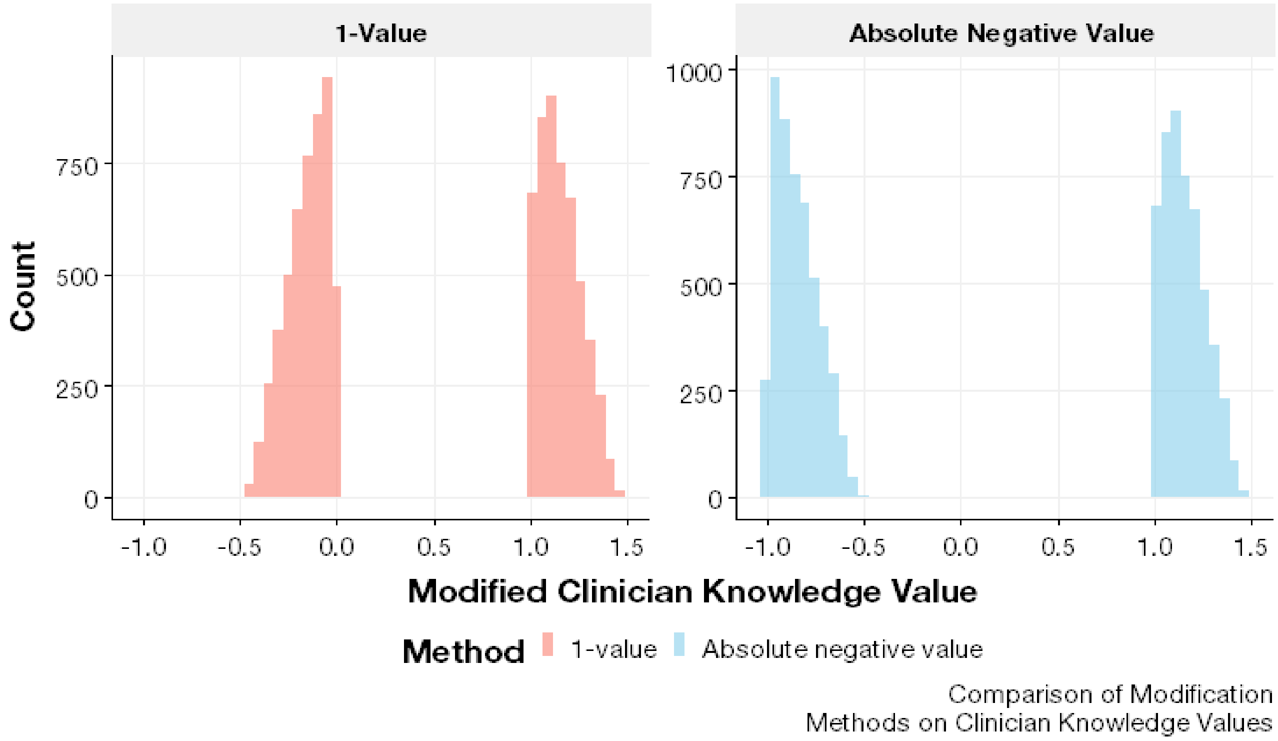

Figure 5 Inversion due to interpreting as decreased risk instead of taking the negative magnitude presented by the odds ratio.

By taking the absolute negative value presented by the risk ratio when $\tau_i < 1$, there is greater penalty as reflected by the decreased score. It would reflect the decrease in risk naturally observed when interpreting the odds ratio (i.e. 0.90 odds ratio would indicate a 10% decrease in risk). For instances scores > 0.90 are more common, it would exercise more conservatism to the detrimental effects of clinician experience and knowledge.

This is the presented justification for using the following.

```
# Education/experience of clinician (estimation)

patients_df$clinknow <- rpert(nrow(patients_df),min = 0.5,

                              mean = 1.0, max = 1.5)



# Modify clinknow values if less than 1

patients_df$clinknow <- ifelse(patients_df$clinknow < 1, -1*(1-patients_df$clinknow),

                               patients_df$clinknow)
```

However, there is the acknowledgement that is this not on a reflect the multiplicative effect that odds ratios represent. The results of this are linear.

Modifications to the distribution as seen by the scenarios will skew the distribution more towards the right.

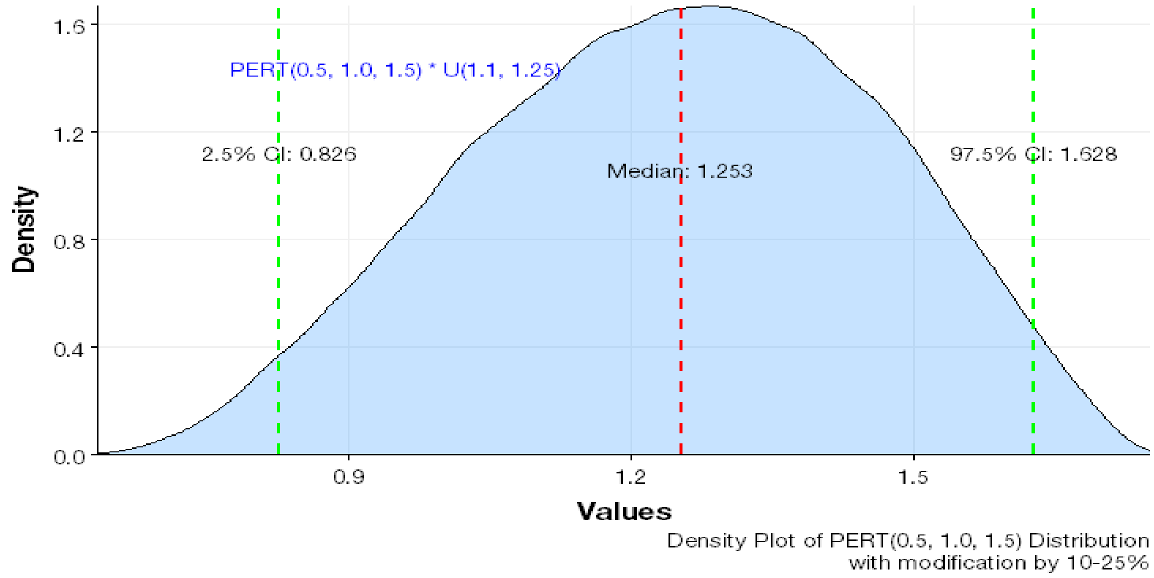

Figure 6 Skewing of distribution with improvements in modifications through random multiplication of a uniform distribution $U\ 1.1,1.25$

Risk conversion patient risk

There is an inherent need to acknowledge similar interpretations of the patient risk. As the main factor modelled is past diagnoses, the odds ratios can be used.

```
# Previous diagnoses
patients_df <- patients_df %>%
  mutate(
    individual_risk = case_when(
      status == "MSM" & diagnoses %in% 0:1 ~ runif(n(), 0.01, 0.10),
      status == "MSM" & diagnoses %in% 2:4 ~ rpert(n(), min = 1.07, mean = 1.59, max =
2.37),
      status == "MSM" & diagnoses %in% 5:9 ~ rpert(n(), min = 0.71, mean = 1.85, max =
4.82),
      status == "MSM" & diagnoses >= 10 ~ rpert(n(), min = 0.636, mean = 2.32, max = 6.55),
      status == "Hetero" & diagnoses %in% 0:1 ~ runif(n(), 0.01, 0.025),
      status == "Hetero" & diagnoses %in% 2:4 ~ rpert(n(), min = 0.37, mean = 0.94, max =
2.37),
      status == "Hetero" & diagnoses %in% 5:9 ~ runif(n(),0.01, 0.10),
      status == "Hetero" & diagnoses >=10 ~ rpert(n(), min = 0.636, mean = 2.32, max =
6.55),
    )
  )
```

In interpreting and utilising the distributions specified, no transformations are needed. When scores are < 1 there is no requirement to interpret as a negative. This has been modelled under the assumptions that 1. *De novo* resistance can occur, thus odds ratios such as 0.01 still denote there is a possibility for resistance regardless of past diagnoses 2. The population modelled are assumed to have a resistant isolate, but the presenting risk differs. 3. In instances such as 5:9 diagnoses in heterosexual males, the proportion of the population for which this reflects is of such a small magnitude. The overall effect on the total proportions of tests initiated is negligible and would model randomised testing. 4. In higher diagnoses, the distribution is largely right skewed which would denote the probability of having low risk negligible.

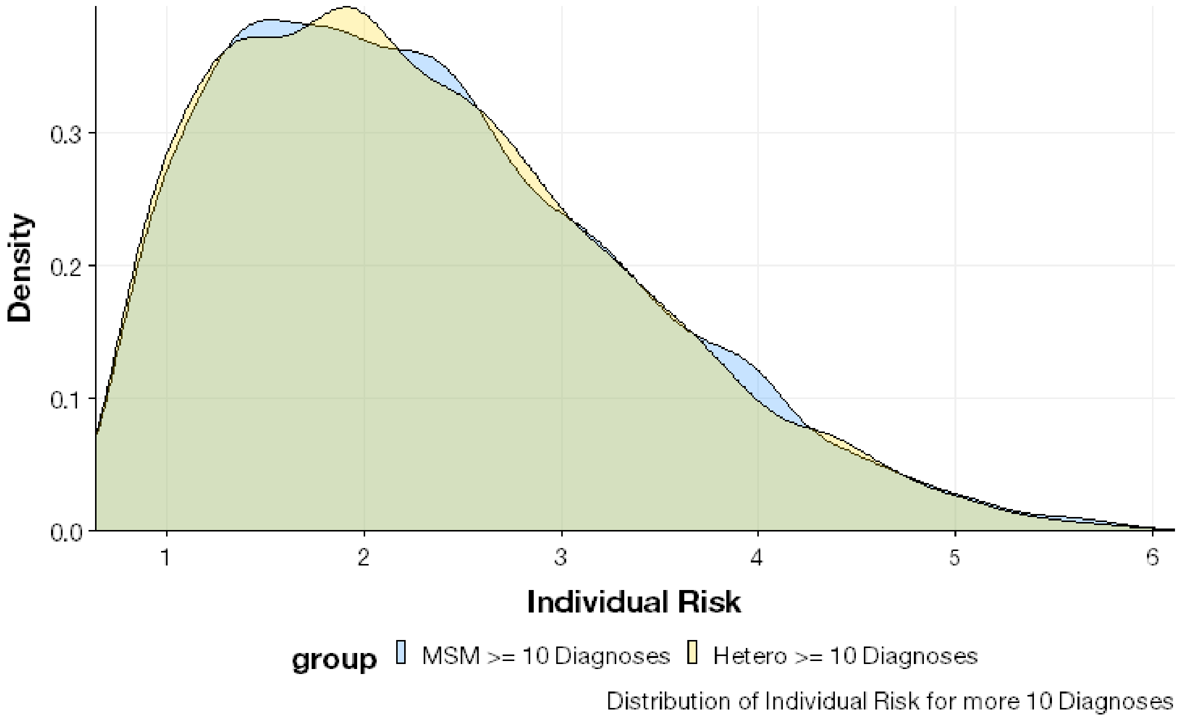

Figure 7 Positive skews within higher diagnoses categories

For the effect of θ_i on the ϕ θ_i value, the following plot demonstrates the change in values with increasing θ_i values.

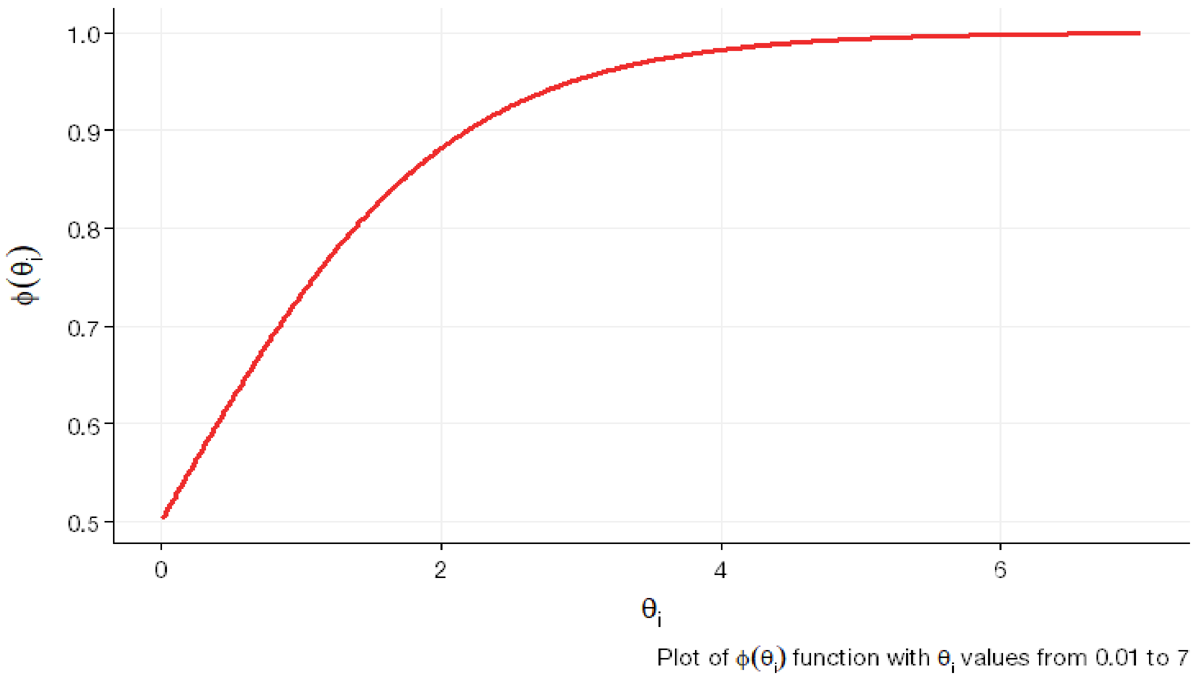

Figure 8 Visualization of the Sigmoid Function $\phi \;\; \theta_i$ Probability Transformation of Individual Risk Score θ_i

A core assumption of this would indicate there is a risk at $\theta_i = 0$ of 0.5 as the baseline risk. This would indicate a degree of randomness within testing (patient could be or could not be a risk, at 50%). This fits within the modelling paradigm for randomness. However, risk does increase as the number of diagnoses, and therefore potential θ_i value.

Mandate Scenario

For mandates, all tests are mandatory for everyone who presents. However, this is of course subject to clinician adherence α. Thus, changes to the system as are as follows:

```
# adherence_value is already assigned based on clinician type

# Modify adherence_values for changes in scenario

  patients_df$policy_test <- sapply(patients_df$adherence_value, function(adherence) {

    # Assigning Probabilistically "test" based on adherence value

    ifelse(runif(1) <= adherence, "test", "no test")

  })

  # After assigning policy_test outcomes checking proportions

  proportion_policy_test_table <- prop.table(table(patients_df$policy_test))
```

Therefore, the modification of the scenarios is presented with the change in adherence proportions and not values. We change the proportion of clinicians which are low/medium/high adherence (can be done in different permutations)

```
# Assigning agents example: Here 5% average, 90% high, 5% low.

patients_df$agent <- sample(c("A", "B", "C"), nrow(patients_df),

                            replace = TRUE, prob = c(0.05, 0.90, 0.05))
```

Sensitivity analysis of binomial testing function

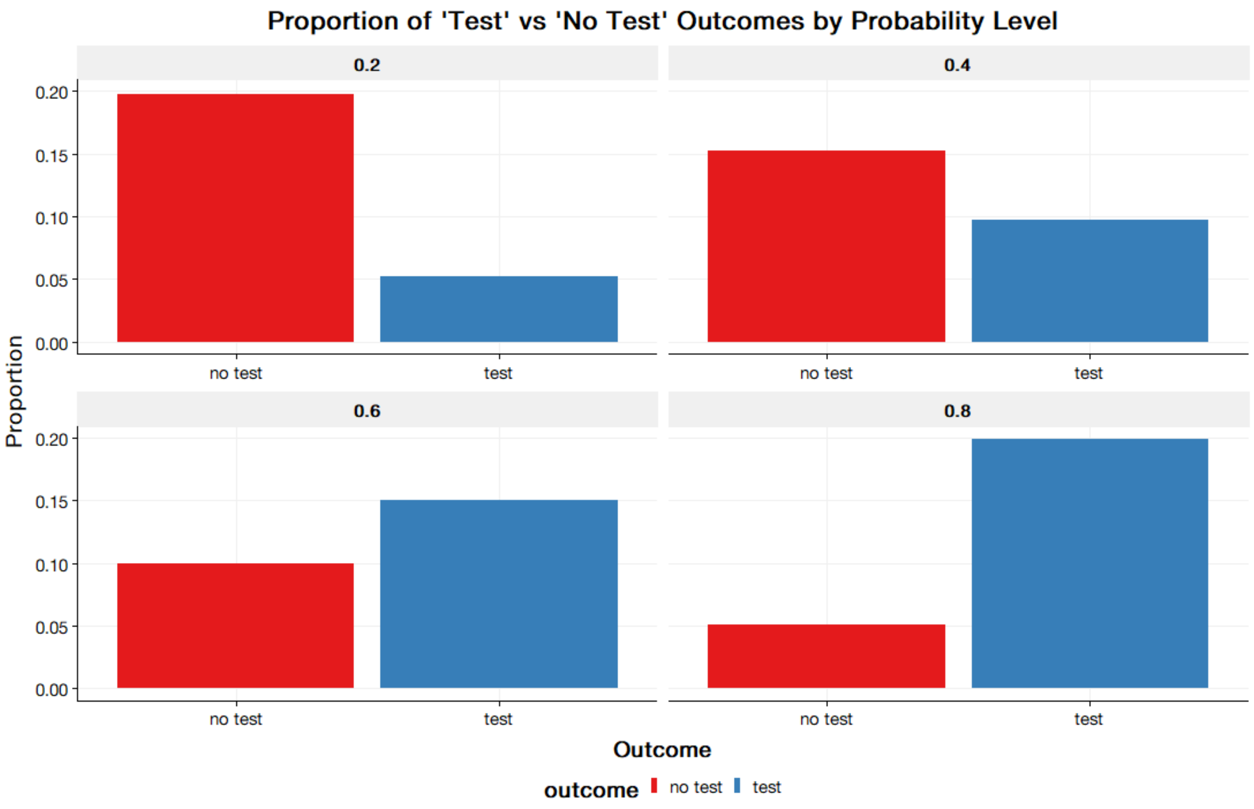

Figure 9 Sensitivity analysis of the binomial testing function with 1000 iterations.

The sensitivity of the testing function $\varphi_i \sim \text{Binomial}\ n, \lambda_i$ is presented in Figure 3. It is expected that over 1000 trials, the proportion of successes aligns with the specified probabilities in the example (0.2, 0.4, 0.6, 0.8)

The model can be found here:

https://github.com/Phu-Do/StochasticHealthSystemsModelAndBBN/tree/main

References

1. Allen H, Merrick R, Ivanov Z, Pitt R, Mohammed H, Sinka K, Hughes G, Fifer H, Cole MJ: **Is there an association between previous infection with Neisseria gonorrhoeae and gonococcal AMR? A cross-sectional analysis of national and sentinel surveillance data in England, 2015-2019**. *Sex Transm Infect.* 2023, **99**(1):1-6,10.1136/sextrans-2021-055298.
2. King J, McManus H, Kwon A, Gray R, McGregor S: **HIV, viral hepatitis and sexually transmissible infections in Australia: Annual surveillance report 2022**. *Australian Government Department of Health and Aged Care.* 202210.26190/sx44-5366.
3. Mauck DE, Gebrezgi MT, Sheehan DM, Fennie KP, Ibanez GE, Fenkl EA, Trepka MJ: **Population-based methods for estimating the number of men who have sex with men: a systematic review**. *Sex Health.* 2019, **16**(6):527-538,10.1071/SH18172.
4. Goyal MK, Witt R, Hayes KL, Zaoutis TE, Gerber JS: **Clinician Adherence to Recommendations for Screening of Adolescents for Sexual Activity and Sexually Transmitted Infection/Human Immunodeficiency Virus**. *The Journal of Pediatrics.* 2014, **165**(2):343-347,10.1016/j.jpeds.2014.04.009.
5. Lugtenberg M, Burgers JS, Besters CF, Han D, Westert GP: **Perceived barriers to guideline adherence: a survey among general practitioners**. *BMC Fam Pract.* 2011, **12**(1):98,10.1186/1471-2296-12-98.
